# Harnessing Extracellular Vesicles for Precision Diagnostics and Infectious Disease Management: Integrating Host‐ and Pathogen‐Derived Cues

**DOI:** 10.1002/exp2.70234

**Published:** 2026-10-30

**Authors:** Ming Zhou, Yongchun Pan, Bo Ning, Christopher J. Lyon, Yang Liu, Tony Hu

**Affiliations:** ^1^ Department of Clinical Laboratory Guangxi Chest Hospital Liuzhou China; ^2^ Center For Cellular and Molecular Diagnostics and Department of Biochemistry and Molecular Biology Tulane University School of Medicine New Orleans USA; ^3^ Department of Hepatobiliary Cancer Liver Cancer Center National Clinical Research Center for Cancer Key Laboratory of Cancer Prevention and Therapy Tianjin Medical University Cancer Institute & Hospital Tianjin's Clinical Research Center for Cancer Tianjin China; ^4^ Department of Hepatobiliary and Pancreatic Oncology Tianjin Cancer Hospital Airport Hospital Tianjin China; ^5^ School of Biomedical Engineering Tsinghua Medicine Tsinghua University Beijing China

**Keywords:** biomarkers, diagnostics, extracellular vesicles, infectious diseases

## Abstract

Conventional infectious disease diagnostics often rely on detecting pathogen‐derived factors or an immune response directed against them to diagnose disease. However, these approaches can lack the specificity to distinguish active and latent infections or pathogenic infections from non‐pathogenic colonization events. Extracellular vesicles (EVs)—nanoscale vesicles that are abundantly secreted by most cells, particularly in response to disease and cell injury—can carry pathogen‐specific factors that indicate the presence and virulence state of a pathogen, as well as immune and inflammatory responses. EVs carrying both types of factors can thus provide valuable information about the presence, identity, and pathogenic phenotype of a microbe in an individual with a suspected infection. Evaluating EVs present in saliva, urine, blood, and other accessible biofluids that contain both host and pathogen information (hpEVs) can thus aid in disease detection, stratification, and monitoring. This review discusses the relevance of EVs to infection and highlights methods that can be used to analyze hpEVs for future clinical applications, including new signal amplification strategies, single vesicle–resolution analysis methods, microfluidic platforms, and AI‐based data analysis approaches. It also discusses key challenges that must be addressed to enable development and adoption of hpEV applications for disease diagnosis and management.

## Graphical abstract

This review summarizes the diagnostic and monitoring potential of host‐pathogen extracellular vesicles (hpEVs) in infectious diseases, advances in their isolation and analysis, and challenges to clinical translation.

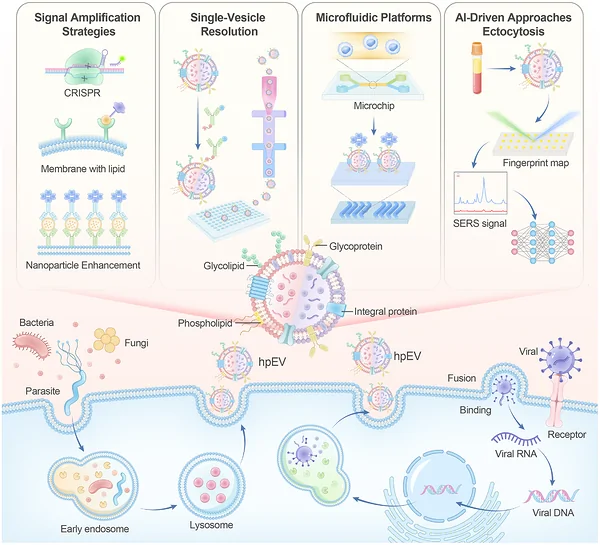

## 1 Introduction

Infectious disease remains a leading cause of morbidity and mortality due to the persistence of several major human pathogens (e.g., tuberculosis [1, 2], malaria [3], and HIV/AIDS [4]), and emerging zoonotic pathogens (e.g., monkeypox virus [5] and the highly pathogenic H5N1 strain of avian influenza [6] have the potential to cause major disease outbreaks. Rapid and sensitive diagnostic strategies are needed to address this issue, since traditional diagnostic approaches (e.g., culture and antigen and nucleic acid tests) that rely on the direct detection of a pathogen or its components [7, 8, 9] often have low sensitivity or specificity, require invasive samples, or have infrastructure requirements that limit their utility in some settings [10, 11].

New diagnostics should also ideally detect latent, asymptomatic, or intracellular infections, particularly in their early stages or in the context of low pathogen burden [12, 13]. Pathogen‐derived factors can be used to detect specific pathogens and sometimes provide information about their virulence. However, there is growing interest in the parallel detection of host‐derived biomarkers that can detect active immune responses, regardless of the pathogen involved [14, 15], to aid in distinguishing active infections from latent infections or colonization and responses to specific treatment regimens.

Extracellular vesicles (EVs), lipid bilayer‐enclosed nanostructures that are abundantly secreted by host cells [16] during disease and associated cell injury processes, carry both pathogen‐ and host‐derived factors that can modulate these processes in their lipid bilayers or lumens. These EV biomolecular cargoes, which consist of host‐ and pathogen‐derived proteins, lipids, nucleic acids, and metabolites, reflect the phenotype of their cell of origin [17, 18] and can provide valuable information about the presence, identity, and behavior of a pathogen affecting them. EVs have thus emerged as a highly informative source of new biomarkers to detect and monitor the progression and treatment responses of specific infectious diseases [19, 20, 21]. Further, since EVs can readily cross most biological barriers and accumulate in most biological fluids, including blood, urine, and saliva, this can permit such analyses to be performed with non‐invasive or minimally invasive specimens [22]—including blood, urine, and saliva—facilitating repeat analyses performed for treatment monitoring and disease management. Notably, EVs also carry and preserve biomolecules that can be rapidly degraded in most biofluids to permit their sensitive and reproducible detection by an array of advanced analytic methods, such as high‐throughput sequencing, proteomics, flow cytometry, and nanoscale imaging, to enable comprehensive molecular profiling of EV subpopulations and signatures from small sample volumes [23, 24].

Both host cells and cellular pathogens can secrete EVs to influence disease development and progression. Host‐derived EVs (hEVs) that accumulate during infection can derive from a variety of cell types, including pathogen‐infected host cells, adjacent but uninfected cells at the infection site, and immune cells recruited to this site. These EVs can thus exhibit distinctive pathogen‐associated composition changes due to the presence of pathogen‐derived factors or changes in the expression of host‐derived factors that are directly regulated by the infectious pathogen or by local or systemic host responses to this pathogen [25]. EVs secreted by host cells during infection can thus be categorized into two classes: EVs released by infected cells, or cells that interact with infected cells, and which thus contain both host‐derived and pathogen‐derived factors (hpEVs); and EVs secreted by uninfected cells or cells that do not significantly interact with them, and thus contain host‐derived factors but not pathogen‐derived factors (hEVs). Finally, cellular pathogens can also directly secrete pathogen‐derived EVs (pEVs) that can transfer factors to adjacent and distant cells to alter their microenvironment and the host immune response. Complex bidirectional host–pathogen interactions regulated by hEVs, hpEVs, and pEVs secreted in response to infection can influence key processes that regulate immune responses, inflammation, virulence, and disease progression [26, 27]. Specific EV‐mediated effects on immune, pathogenic, and recovery responses induced after pathogen exposure [28, 29] have been revealed by advances in multi‐omics profiling, single‐vesicle analysis, and high‐sensitivity detection platforms [30, 31, 32] over the past decade.

Based on these findings, this Review is unique in conceptually dividing infection‐related extracellular vesicles into three distinct subgroups: hEVs, pEVs, and hpEVs (Figure 1). Unlike previous reviews focusing on EVs (including those specifically addressing infectious diseases), which typically treat EVs as a broadly unified group, this framework allows for clearer differentiation of the diverse biological information carried by EVs during infection. Notably, hpEVs integrate pathogen‐derived components and host response signals, constituting a highly informative biomarker source that provides insight into host–pathogen interactions and the EV‐associated factors capable of modulating the behavior of recipient cells. Several emerging technologies have also facilitated the sensitive detection and high‐resolution analysis of hpEV populations, including new signal amplification techniques, single vesicle resolution approaches, and AI‐mediated data analysis methods. Collectively, these tools are serving to accelerate the bench‐to‐bedside translation of EV research by supporting the development of new sensitive high‐throughput methods to diagnose and evaluate specific infectious diseases.

**FIGURE 1:**
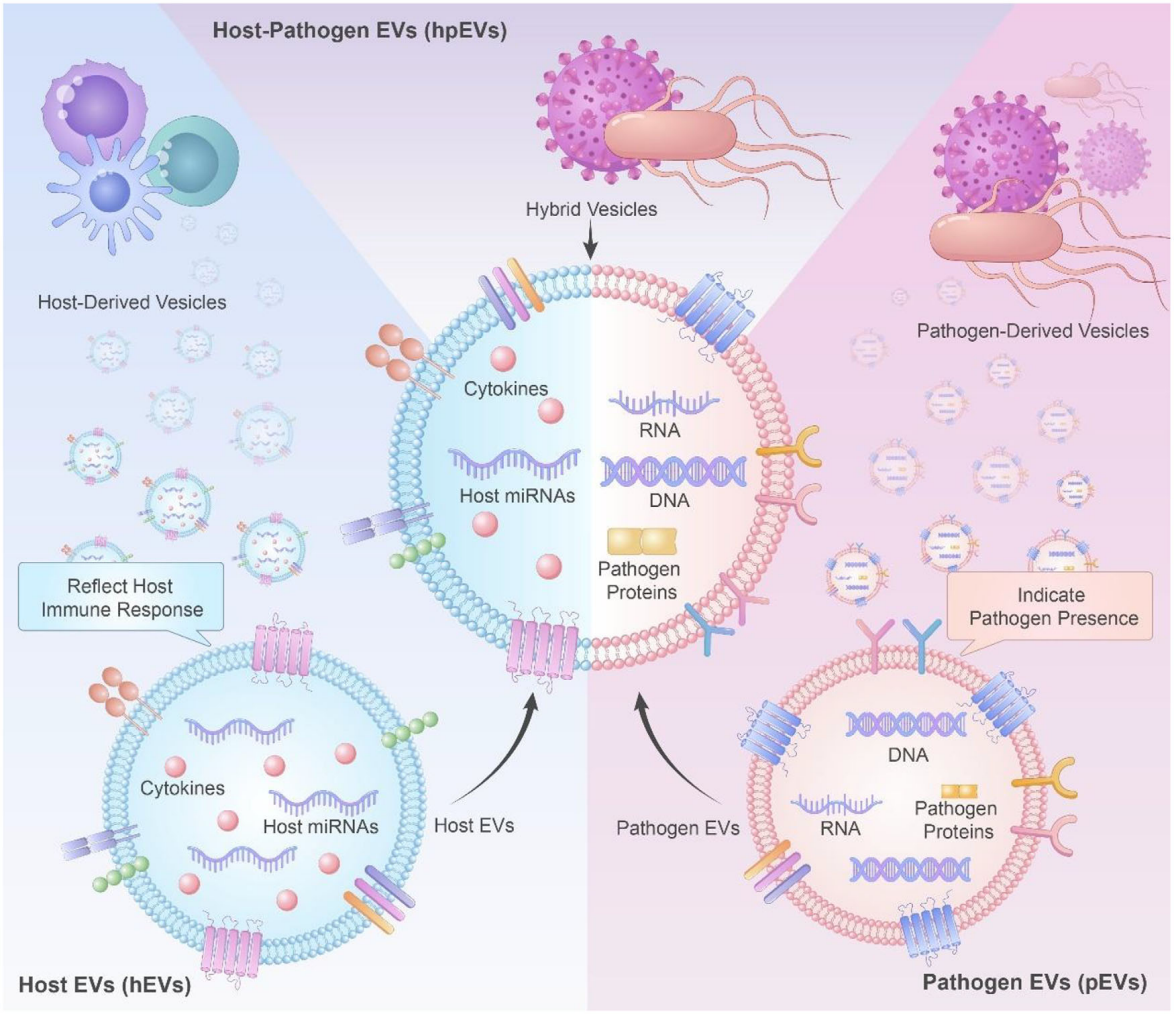
Conceptual overview of host‐derived, pathogen‐derived, and host–pathogen hybrid extracellular vesicles in infectious diseases. Host‐derived EVs (hEVs) reflect the host physiological state. Pathogen‐derived EVs (pEVs) are released by pathogens and indicate pathogen presence through pathogen‐specific molecules. Host–pathogen hybrid EVs (hpEVs) originate from infected host cells and combine host and pathogen signals, providing integrated information on infection status.

In this review, we therefore highlight the benefits of hpEV‐based disease diagnostics and discuss the emerging technologies that promise to facilitate their development and clinical translation, as well as the relative benefits of using hpEVs instead of pEVs for disease diagnosis and evaluation. We first discuss the complementary information provided by hEVs and pEVs and how analyzing host‐derived and pathogen‐derived EV components in hpEVs can provide additional benefits. We next discuss emerging technologies and platforms that promise to facilitate the detection and analysis of these EVs, as well as key translational challenges for hpEV‐based assays, and identify areas that warrant additional future study.

## 2 EVs Regulate Processes That Control Disease Pathogenesis

EVs mediate critical interactions between pathogens and the host immune system [33] since hEVs and pEVs can collaboratively remodel the immune microenvironment to play a significant role in the onset and progression of disease [29]. Host‐derived EVs reflect the host's physiological and immune status, including inflammation, tissue damage, and immune activation [34]. Pathogen‐derived EVs, on the other hand, carry the infecting agent's molecular signatures, such as pathogen‐specific nucleic acids or proteins, providing direct evidence of pathogen presence and virulence. Analyzing host‐ and pathogen‐derived components simultaneously—in hpEV populations—offers a more comprehensive view of host–pathogen interactions, thereby enhancing diagnostic sensitivity, enabling better disease stratification, and potentially informing targeted therapeutic strategies (Figure 2 and Table 1). In this section, we therefore discuss the complementary roles of host‐ and pathogen‐derived EV components in the development and pathogenesis of select infectious diseases and the application of data derived from hEVs and pEVs.

**FIGURE 2:**
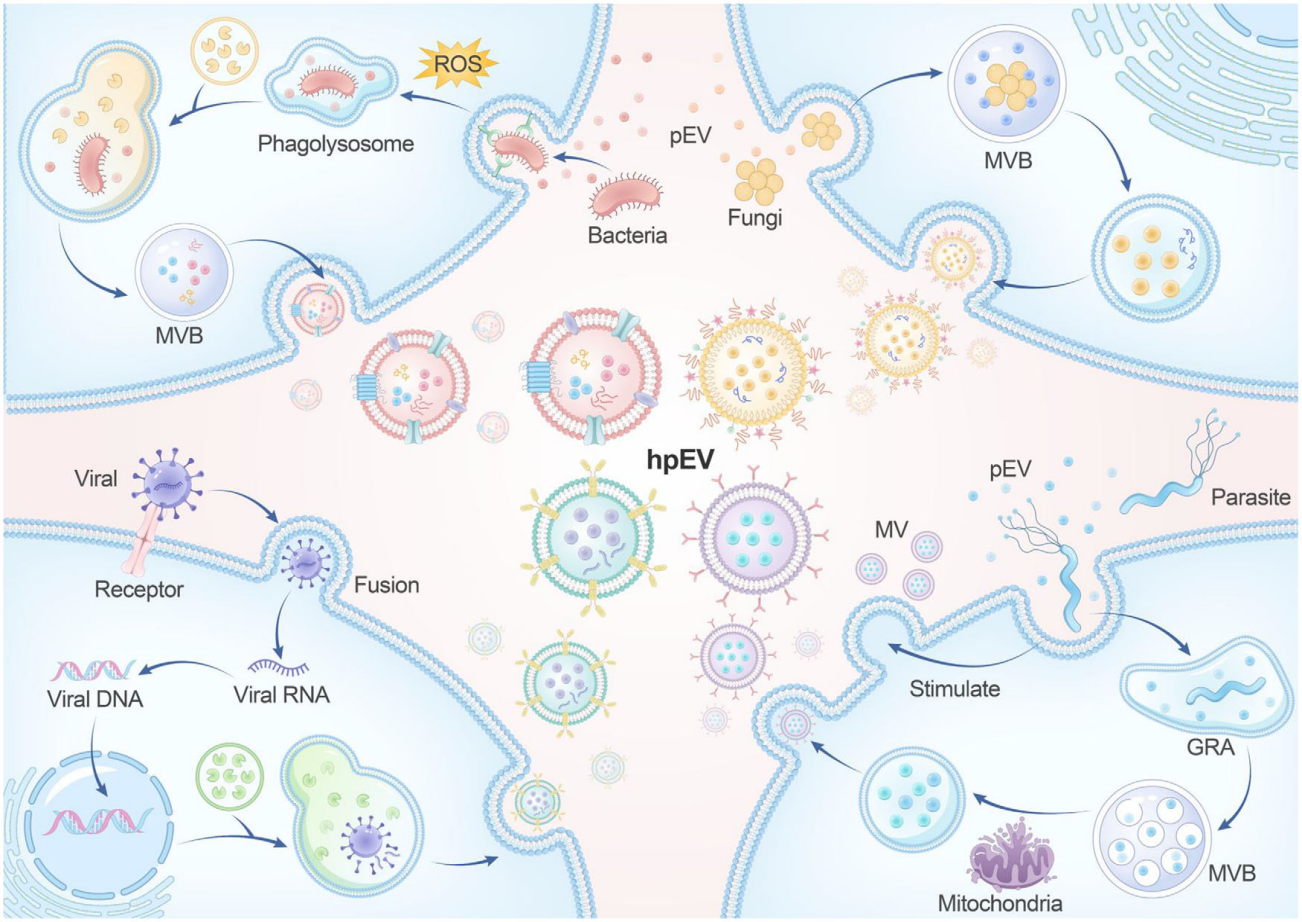
Schematic of the biogenesis and release of pathogen‐derived extracellular vesicles (pEVs) and host‐derived extracellular vesicles (hpEVs). After invading host cells, bacteria, fungi, viruses, and parasites interact with organelles such as lysosomes, multivesicular bodies (MVBs), and mitochondria, triggering the exosomal pathway. This process leads to the generation and release of hpEVs that contain pathogen proteins or nucleic acids. Meanwhile, pathogens themselves can secrete pEVs carrying virulence factors or genomic fragments, contributing to host–pathogen communication and the progression of infection.

**TABLE 1:** Comparison of hEVs, pEVs, and hpEVs in infectious disease diagnostics.

Category	hEVs	pEVs	hpEVs
Definition	EVs released from host cells under physiological or pathological conditions	EVs secreted directly by pathogens (bacteria, viruses, fungi, parasites)	EVs derived from host cells but containing pathogen‐derived components
Cellular origin	Host cells (e.g., immune cells, epithelial cells)	Pathogens (e.g., Mycobacterium, viruses, bacteria)	Host cells interacting with pathogens (e.g., infected macrophages)
Biogenesis	Endosomal pathway or plasma membrane budding in host cells	Pathogen‐specific secretion mechanisms	Host EV biogenesis modulated by pathogen infection
Cargo composition	Host proteins, lipids, RNAs, metabolites	Pathogen proteins, nucleic acids, virulence factors	Mixed host and pathogen molecules (proteins, RNAs, lipids, antigens)
Molecular signatures	Host biomarkers (e.g., CD9, CD63, CD81, miRNAs)	Pathogen‐specific markers (e.g., bacterial proteins, viral RNAs)	Combined host and pathogen signatures
Immunological role	Immune regulation, intercellular communication	Virulence, immune evasion, pathogen–host communication	Immune activation, antigen presentation, inflammatory signaling
Abundance in biofluids	High abundance	Generally low abundance	Intermediate abundance
Specificity for infection	Low–moderate	High	Very high
Stability in circulation	High	Moderate–low	High
Diagnostic sensitivity	Moderate	Low–moderate	High
Diagnostic specificity	Low–moderate	High	Very high
Clinical relevance	Reflect host response but lack pathogen specificity	Direct evidence of pathogen presence but limited detectability	Integrated information on pathogen presence and host response
Diagnostic implication	Useful for disease monitoring and prognosis	Useful for pathogen identification	Optimal biomarkers for infection diagnosis and stratification
References	[27, 35, 36, 37, 38, 39, 40, 41, 42, 43]	[44, 45, 46, 47, 48, 49, 50, 51, 52, 53, 54, 55]	[42, 56, 57, 58, 59, 60]

### 2.1 Effects of pEVs Factors on Disease Pathogenesis and the Host Response

Bacteria, fungi, and parasites that cause human disease can secrete EVs that influence the host response to infection, and some viruses have also been reported to hijack the EV biogenesis pathway to generate pEVs. Despite their diverse origins and modes of biogenesis, pEVs from various pathogens have been shown to modulate host innate and adaptive immunity by delivering pathogen‐associated molecules—such as RNA, enzymes, or virulence factors—into host immune or epithelial cells [56]. These molecular cargos can alter intracellular signaling pathways, suppress antigen presentation, dampen pro‐inflammatory responses, or promote an immune‐permissive environment to facilitate evasion of the immune response and promote infection and disease progression [61]. Here, we summarize the major effects of pEVs derived from different types of pathogens.

#### 2.1.1 Bacteria‐Derived EVs

Bacteria generate pEVs via diverse pathways to regulate host responses and enhance bacterial survival, dissemination, and chronic infection states [16]. Bacteria actively release pEVs via tightly regulated biogenetic pathways [62]. Both gram‐positive and negative bacteria release membrane vesicles (MVs), but only gram‐negative species release outer membrane vesicles (OMVs) [63]. MVs are generated via mechanisms that employ localized remodeling of the bacterial cell wall and turgor‐driven protrusion of the bacterial membrane, and OMVs derive from the controlled bulging and pinching‐off of the outer membrane, which is often triggered by envelope stress, misfolded proteins, or quorum‐sensing signals [64]. MVs and OMVs also exhibit distinct molecular composition and functional properties. OMVs are enriched in lipopolysaccharide, outer membrane proteins, and periplasmic components, while MVs—particularly from Gram‐positive bacteria—lack LPS but carry surface proteins, cytoplasmic enzymes, and toxins. These compositional differences underlie their distinct roles in virulence and host–microbe interactions. In Mycobacterium tuberculosis, MVs are enriched in lipoarabinomannan (LAM), a glycolipid that impairs macrophage function by suppressing inflammatory signaling, inhibiting phagosome maturation, and interfering with antigen presentation—ultimately facilitating immune evasion [44]. In *Salmonella enterica* bacilli, OMV production is modulated by their PhoPQ two‐component system, which also controls LPS remodeling and upregulates the expression of the PhoP‐activated gene C protein, which can facilitate immune evasion by recruiting complement factor H [45]. MVs and OMVs are selectively loaded with lipoproteins, toxins, nucleic acids, peptidoglycan fragments, and other factors via processes governed by specific cargo‐sorting machinery and the organization of their protein–lipid microdomains [65]. Several of these factors exert known effects on host cells and responses. For example, OMVs secreted by *Escherichia coli* and *Neisseria meningitidis* can deliver LPS to the cytosol, where it activates caspase‐11 and triggers pyroptosis via the non‐canonical inflammasome pathway—an essential innate immune mechanism in the response to Gram‐negative bacterial infections. OMVs derived from enterohemorrhagic *E. coli* (EHEC) O157 can also strongly induce intestinal epithelial cell pro‐inflammatory responses by stimulating IL‐8 production via the action of OMV‐associated virulence factors, including H7 flagellin and O157 LPS, which respectively induce TLR5 and the TLR4/MD‐2 signaling to activate NF‐κB–dependent transcriptional responses [46, 47]. Environmental stress can also alter the biogenesis and composition of these vesicles. For example, oxidative stress and antibiotic exposures can enhance vesicle release by *Staphylococcus aureus* bacilli and result in their enrichment with α‐toxin and superantigens that can disrupt plasma membrane integrity and host hyperactivate pattern‐recognition receptor (PRR) pathways involved in pro‐inflammatory responses. Bacterial pEVs can thus not only carry bioactive pathogenic factors that can serve as diagnostic biomarkers, but also factors that may indicate the physiologic state of the bacteria that secreted them, which could potentially serve as prognostic biomarkers in applications designed to monitor the disease responses to treatment interventions [66].

#### 2.1.2 Fungus‐Derived EVs

Pathogenic fungi such as *Cryptococcus neoformans*, *Candida albicans*, and *Aspergillus fumigatus* also release pEVs loaded with diverse cargoes of immunomodulatory factors—including mannoproteins, lipid moieties, small RNAs, and virulence factors (e.g., secreted aspartyl proteases and polysaccharides such as glucuronoxylomannan [48])—that can facilitate immune evasion and fungal persistence [48, 49]. EVs secreted by fungi belonging to the genus *Cryptococcus* inhibit phagocytosis and T‐cell activation, while those secreted by *C. albicans* can promote host tissue degradation and contribute to biofilm development [50, 51, 67]. Fungal RNAs associated with pEVs have been implicated in reprogramming host gene expression, leading to suppressed proinflammatory responses and the promotion of fungal tolerance. Candida albicans pEVs deliver fungal DNA into the macrophage cytosol, activating the cGAS–STING pathway and downstream IRF3/TBK1 signaling to induce type I interferon responses [50]. This non‐canonical pathway bypasses dectin‐1/CARD9 and TLR9 signaling and increases host susceptibility, as cGAS‐ or STING‐deficient mice show improved survival. Together, these pathways—including the IL‐12–JAK2–STAT4 axis for TH1 polarization and the IFN‐γ–JAK1/JAK2–STAT1 axis for macrophage activation—constitute the core of type I immune responses that drive intracellular fungal clearance. These coordinated responses are particularly critical for controlling pathogens such as *Cryptococcus* and *Histoplasma*, which replicate within macrophages and evade extracellular defenses, thereby underscoring the importance of understanding how fungal pEVs may modulate these cell‐intrinsic antimicrobial mechanisms [52]. These findings thus indicate that fungal pEVs can regulate innate immunity [50].

#### 2.1.3 Virus‐Associated EVs

Although viruses themselves do not secrete EVs, some viruses have evolved sophisticated approaches to hijack pathways that regulate the endosome, multivesicular body, and autophagy pathways of their host cells to cause them to release vesicles that can serve as Trojan horses [68] by masking viral components within a host‐derived lipid bilayer to attenuate immunity and facilitate viral spread [69]. Such EVs can mimic host‐derived exosomes (small ≈30 to 150 nm diameter EVs of endosomal origin) that play essential roles in intercellular communication and subvert immune surveillance. EV cargoes of infected cells can incorporate viral RNAs, proteins, and sometimes complete infectious particles. Non‐enveloped viruses such as hepatitis A virus and enterovirus 71 can employ the EV biogenesis pathway to secrete “quasi‐enveloped” virions so that virions encapsulated within EV lipid membranes are shielded from neutralizing antibodies to promote intercellular transmission of these cloaked viruses and persistent infections [70]. Similarly, enveloped viruses, including Zika virus [71] and members of the hepatitis virus family [72], can utilize the EV biogenesis machinery of their host cells to package their viral particles, genomic material, or other factors to escape immune detection during cell‐to‐cell transmission or alter the virus‐directed immune response. For example, EVs from hepatitis B‐ and C‐infected cells suppress antiviral immunity and enhance viral replication by transferring interferon‐modulating and RNA‐stabilizing factors [73]. EVs released by virus‐infected cells can also modulate signaling via innate immune pathways in uninfected cells [74]. Some of these vesicles stimulate antiviral pathways; for instance, EVs released by hepatitis C virus‐infected hepatocytes can activate TLR7 signaling in plasmacytoid dendritic cells to trigger interferon responses [75]. However, EVs released by virus‐infected cells can also attenuate antiviral immune responses by transferring miRNAs that inhibit interferon production. EVs derived from enterovirus 71‐infected cells can deliver miR‐30a to inhibit MyD88 expression and its downstream signaling activity, while EVs secreted by cells infected with Newcastle disease virus are enriched in miR‐198, miR‐1184, and miR‐1273f, all of which contribute to reduced IFN‐β expression in EV recipient cells [76, 77]. Epstein–Barr virus and other DNA viruses can also exploit the host EV system to export viral RNAs and immunomodulatory proteins that reprogram recipient cells towards more permissive states [78], by interfering with their PRR signaling responses and enhancing viral replication. Virus‐mediated changes in EV composition can thus influence an array of host processes that influence viral transmission, replication, and persistence, and these EV changes can thus serve as potential biomarkers of these processes to improve disease diagnosis and management.

#### 2.1.4 Parasite‐Derived EVs

Protozoan parasites and helminths have evolved sophisticated strategies that employ their EVs to alter cell responses that can promote persistent infections through their influence on various host processes [79]. For example, in one well‐studied example, EVs secreted by the parasitic helminth *Heligmosomoides polygyrus* transfer small RNAs—primarily microRNAs (miRNAs) and Y RNAs—into host cells to regulate host immune pathways, and particularly by suppressing inflammatory responses associated with type II hypersensitivity. This helps the parasite evade immune‐mediated clearance [53]. Similarly, *Trypanosoma brucei* releases EVs that play critical roles in modulating host–parasite interactions and immune responses. These EVs are derived from membrane nanotubes and are capable of mediating communication between parasites by transferring the serum resistance–associated (SRA) protein. SRA is essential for the parasite's resistance to trypanolytic factors present in human serum. Additionally, these EVs can fuse with host red blood cells, delivering lipids and proteins that compromise red cell integrity and contribute to anemia, a common pathological consequence of trypanosome infection [54]. EVs derived from the helminth *Ascaris* have also been shown to exert potent immunomodulatory effects. These EVs can enter human monocytes and reprogram them toward a regulatory, anti‐inflammatory phenotype characterized by CD14^+^, PD‐L1^+^ expression, and loss of CCR2. These altered monocytes exhibit suppressed T cell activation, contributing to an immunosuppressive environment favorable to the parasite. Moreover, in mouse models of intestinal inflammation, systemic injection of *Ascaris* EVs has been shown to reduce gut damage, suggesting their potential for therapeutic application in immune‐mediated diseases [55]. Parasite‐derived EVs are thus powerful functional agents that can regulate a wide range of disease‐associated host processes, including immune signaling pathways, cytokine secretion, metabolic regulation, antigen presentation, and tissue remodeling, which can contribute to parasite persistence, immune evasion, and disease progression.

### 2.2 Utility of Pathogen‐Derived EVs for Disease Diagnosis and Evaluation

Pathogen‐derived EVs can contain factors that identify the specific pathogen responsible for an infection and its viability. Additionally, the transfer of factors by pEVs can regulate the host response and disease pathology. Therefore, pEVs hold considerable promise as a source of biomarkers for new diagnostic and prognostic applications [80, 81]. Such biomarkers could in theory evaluate pathogen responses in near real‐time, improve differential diagnosis of diseases with similar clinical presentations, and facilitate longitudinal monitoring of disease progression and pathogen responses to specific therapeutic interventions.

However, applications that employ pEV‐biomarkers must distinguish these EVs from diverse hEV populations and other non‐vesicular components, which are usually much more abundant in diagnostic specimens. Since pEVs may be present at very low concentrations relative to the diverse and abundant host‐derived EV populations in clinical specimens, it may be necessary to selectively enrich these pEVs in some cases to prevent their biomarker signals from being masked by the background of the overall EV population. Further, depending on the pathogen and sample type, pEVs may need to be distinguished from EVs derived from other non‐pathogenic organisms that are also present in the same sample. Some diagnostic specimens may also contain EVs derived from non‐pathogenic microbes or derived from microbes at other anatomical sites. Stool specimens represent an extreme example of this issue, but saliva and sputum specimens are also not aseptic and can contain variable levels of EVs derived from microbes present in these specimens or derived from other sites.

Analysis of pEV biomarkers may therefore require the isolation of these EVs from the general EV population of a specimen prior to analysis, or the use of an analytic approach that can directly distinguish these specific pEV biomarkers from factors contributed by other more abundant EVs present in a specimen. Since the physical characteristics of pEVs are frequently very similar to those of hEVs, affinity enrichment approaches that target surface‐expressed pEV‐biomarkers frequently represent the most feasible means of pEV enrichment.

Several approaches have been explored for their ability to selectively enrich or detect pEVs in complex specimens. These include affinity enrichment methods employing a diverse array of affinity materials (e.g., antibodies, aptamers, molecularly imprinted polymers [82]) that recognize pEV‐specific factors, or probes specific for pEV biomarkers. LPS is a common target for both EV enrichment and detection methods, with one group using it as a molecularly imprinted polymer target (Figure 3A–C) and another using it as the ligand for a fluorescently conjugated antibiotic probe [66] (Figure 3D).

**FIGURE 3:**
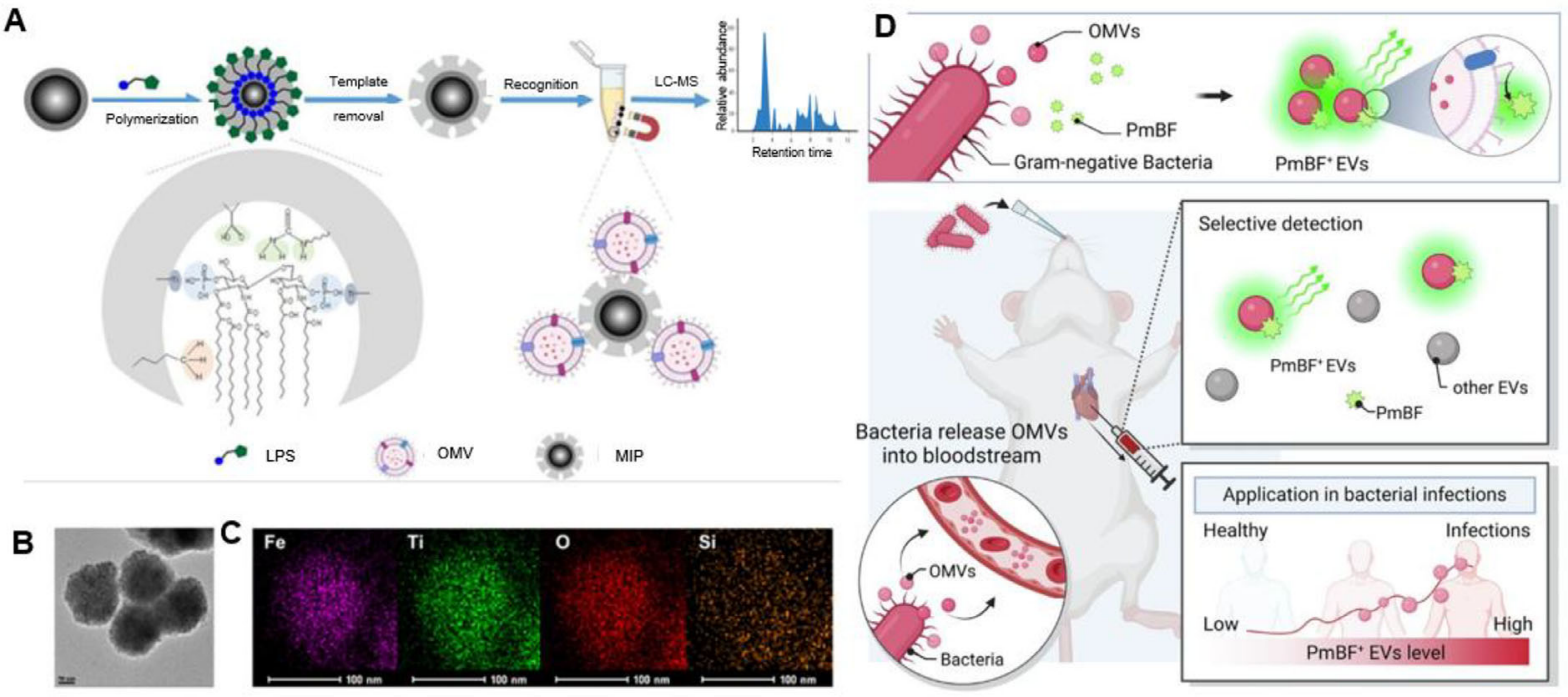
Strategies for the isolation and detection of pathogen‐derived extracellular vesicles (pEVs). (A–C) Molecularly imprinted polymer (MIP). (A) Schematic illustration of the MIP synthesis process. (B) Transmission electron microscopy image and (C) Energy‐dispersive X‐ray spectroscopy (EDX) mapping, confirming the morphology and elemental composition of MIP. Reproduced with permission [82]. Copyright 2024, American Chemical Society. (D) Antibiotic‐conjugated probe (PmBF) specifically labels outer membrane vesicles (OMVs) for early detection of bacterial infections in blood. Reproduced with permission [66]. Copyright 2025, Nature Publishing Group.

These pEV assay approaches, or related ones, have the potential to streamline the diagnosis of select infectious diseases that are difficult to rapidly or accurately diagnose by conventional approaches. Such pEV‐based assays could have particular utility for early disease detection, assessment of disease burden, and longitudinal monitoring of disease progression and pathogen responses to treatment intervention. However, new pEV‐based diagnostics also face significant challenges due to the issues above, including low pEV concentrations in diagnostic samples, particularly early in disease development; and the potential for pEV signal to be masked by background derived from more abundant hEVs, or EVs from non‐pathogenic microorganisms, particularly in non‐sterile specimens in the absence of effective selective enrichment or detection approaches. In this context, hEV and biomarker results have emerged as attractive complementary data to support pEV assay findings, and hpEV results may serve as an alternative means for direct disease diagnosis and evaluation, due to their greater relative abundance of these EVs and their ability to reflect host immune responses and pathogen‐induced changes in these and other disease processes, as explored in the following section.

### 2.3 Effects of Host‐Derived EVs on Disease Pathogenesis and the Host Response

Several characteristics of hEVs render them particularly valuable as a source of biomarkers for disease diagnosis, prognosis, and treatment evaluation. EVs derived from infected cells and cells affected by an infection are often abundant in a diverse array of biospecimens, much more so than pEVs; can be readily obtained using non‐invasive or minimally invasive procedures; and can provide a comprehensive snapshot of a patient's pathologic responses to infection. Host‐derived EVs can carry factors that reflect local and systemic responses induced upon infection, including those that can regulate pathogen‐induced immune and pro‐inflammatory responses and tissue injury and remodeling pathways. Following infection, multiple cell types—including epithelial barrier cells, innate immune cells, and other cells—can rapidly upregulate EV biogenesis and package EV cargoes with altered immunoregulatory functions. These EVs can be broadly categorized as those directly derived from cells infected by intracellular pathogens—including all viruses and some bacteria, fungi, and parasites—and EVs secreted by cells affected by factors released by a pathogen or its host cell. Changes in these EV cargoes can provide information about systemic and local phenotypes induced upon infection—or upon treatment intervention—but can lack pathogen specificity depending on whether they derive from infected or non‐infected cells. Nevertheless, information derived from both categories of EVs can still prove valuable for disease diagnosis and management.

#### 2.3.1 EVs Secreted by Non‐Infected Cells

Non‐infected immune cells can actively secrete EVs that play critical roles in coordinating both local and systemic immune responses to infection [35], as they can carry an array of factors with immunologic activity, including cytokines, chemokines, antigen‐presenting protein complexes, costimulatory proteins, and regulatory RNAs [36]. For example, EVs derived from dendritic cells can present pathogen‐associated factors to enhance T cell activation, while EVs released by activated T cells may modulate antigen‐presenting cell functions or influence B cell maturation [37]. Neutrophil‐derived EVs often contain antimicrobial peptides and enzymes that contribute to pathogen clearance and modulate adjacent cell behavior [38]. These EVs can either promote immune activation or facilitate the resolution of inflammation, depending on the context of the infection.

Non‐immune cells, including epithelial, endothelial, and stromal cells, can also secrete EVs in response to pathogen exposure or inflammatory stimuli. These vesicles can influence vascular permeability, leukocyte recruitment, and tissue barrier integrity. For example, epithelial cell‐derived EVs may carry chemotactic signals that guide immune cells to mucosal surfaces, while endothelial cell‐derived EVs can contribute to pro‐inflammatory and pro‐coagulant states, particularly in severe infections.

Further, EVs secreted by both immune and non‐immune cells frequently contain factors that serve as pathogen‐ and damage‐associated molecular patterns (PAMPs and DAMPs); pattern‐recognition receptors (PRRs, such as TLR2, TLR4, NOD1, and NOD2) that recognize these PAMPs and DAMPs; proinflammatory cytokines; chemokines; antimicrobial peptides; and major histocompatibility complexes (MHCs) that contain pathogen‐derived peptides [27, 36].

Host‐derived EVs can also function as “molecular sponges” that bind and sequester pathogen‐derived virulence factors and other factors associated with the disease process to attenuate its progression and pathogenesis. Such EVs can selectively express a variety of surface factors, including ADAM10, CD14, and glycosaminoglycan motifs, that bind bacterial toxins such as α‐toxin or LPS to reduce their bioavailability [39, 40, 41, 42]. Neutralization of these virulence factors by these hEVs can mitigate tissue damage and systemic inflammatory responses. Such protective hEV “sponges” can be actively released by the host during the course of an infection as part of a self‐defense mechanism [43]. Surface proteins and other factors enriched on these EVs can thus reflect the status of the host's immune response, and the presence and abundance of these EV subsets could serve as indicators of host–pathogen interaction dynamics and infection severity.

#### 2.3.2 EVs Secreted by Infected Cells

Cells infected with intracellular pathogens can exhibit profound changes in their secretory behavior, particularly with regard to their EV secretion. Both infected immune and non‐immune cells can release EVs enriched in immunomodulatory factors that reflect the activation of cell stress, innate immunity, or pathogen‐driven responses. However, these EVs can also carry pathogen‐derived factors (e.g., nucleic acids, proteins, lipids), and host‐derived factors (pro‐inflammatory molecules (e.g., IL‐1β, TNF‐α, and type I interferons), chemokines, and antigen‐presenting complexes loaded with microbial peptides). Such hpEVs can interact with various cell populations to modulate immune surveillance, promote or dampen inflammation, influence immune evasion, or exacerbate tissue pathologies. Pathogen‐derived factors may be randomly incorporated into nascent vesicles during normal EV biogenesis, but some infections can activate cell mechanisms that enhance EV secretion and promote the inclusion of pathogen‐specific components or immune modulatory molecules [57] to actively alter the activity of the EVs secreted by their host cells. Cell stress mechanisms and membrane perturbations can contribute to aberrant incorporation of pathogen‐derived factors or host biomolecules into the EVs secreted by infected cells. However, some pathogens can also actively manipulate EV biogenesis and express proteins that alter EV sorting mechanisms to promote the preferential packaging of specific molecular signatures. EVs secreted by infected cells can thus provide substantial information about the phenotype of the pathogen, as well as the infected cells, which may be useful in evaluating the state of an infection, predicting disease severity, and evaluating or predicting treatment outcomes.

##### 2.3.2.1 EVs Secreted by Pathogen‐Infected Immune Cells

During infection, immune cells—particularly macrophages—undergo profound phenotypic reprogramming, leading to the release of EVs that influence the surrounding immune landscape [83, 84]. Infected macrophages can secrete EVs enriched with inflammatory cytokines, chemokines, and signaling mediators that shape both local and systemic responses [56]. These vesicles can activate bystander immune cells, including dendritic cells, T cells, and neutrophils, to promote their recruitment, maturation, or cytokine secretion. For example, in an experimental model of infection, inflammasome‐activated macrophages have been shown to release EVs that induce NF‐κB signaling in neighboring cells to enhance their pro‐inflammatory gene expression and contribute to a feed‐forward inflammatory phenotype loops [85, 86]. Such hyperactivation events may lead to collateral tissue damage in certain contexts. Similarly, pyroptotic immune cells can also release EVs containing a pore ‐forming protein (gasdermin D) that, when transferred to adjacent cells, can induce secondary pyroptosis events that intensify the pro‐inflammatory response and disrupt tissue homeostasis at the affected site [42]. These findings underscore the role of EVs as both immunostimulatory messengers and potential drivers of pathological inflammation during infection.

EVs secreted by infected cells, especially macrophages, frequently exhibit enrichment for pathogen‐derived factors, and PAMPs carried by these EVs can be recognized by pattern recognition receptors (PRRs) on recipient immune cells to facilitate pathogen‐induced immune activation responses [70]. Similarly, dendritic cells exposed to EVs containing pathogen‐derived factors can upregulate their expression of major histocompatibility complex and costimulatory proteins to enhance their antigen presentation activity and ability to activate pathogen‐specific T cell responses. These EVs may therefore contribute to the dissemination of pathogen‐derived antigens and prime the systemic immune response. However, some intracellular pathogens can hijack the EV secretion pathway of their host cells to evade host immunity by altering their EV cargos to suppress interferon signaling or skew the polarization of immune cells toward regulatory or tolerogenic phenotypes. EVs secreted by infected immune cells can thus serve as vectors for pathogen‐derived or pathogen‐induced factors that can promote either pathogen clearance or the suppression of a pathogen‐directed immune response.

##### 2.3.2.2 EVs Secreted by Other Pathogen‐Infected Cell Types

Non‐immune cell types, including epithelial cells, hepatocytes, and neurons, can also actively regulate host–pathogen interactions by secreting EVs that contain pathogen‐derived factors. Following infection, these cells can undergo profound transcriptional and post‐transcriptional changes, resulting in the release of EVs enriched with immunomodulatory RNAs, proteins, or pathogen‐derived factors. These EVs can regulate immune cell phenotypes and alter local or systemic inflammation to contribute to antimicrobial defense or facilitate pathogen persistence. For example, bladder epithelial cells infected with uropathogenic *E. coli* bacilli secrete miR‐18a‐5p‐enriched EVs that can attenuate macrophage PTEN expression and activate the MAPK/JNK signaling pathway [58] to impair phagocytosis and increase TNF‐α secretion and apoptosis. This can compromise the local immune defense and allow bacterial persistence in the urinary tract.

Several viruses and prion‐based diseases also employ EVs secreted by their host cells as transmission vectors. In prion disorders, neurons or glial cells can secrete EVs that contain a misfolded prion protein that seeds the misfolding of this protein in EV recipient cells, in a process that can accelerate the spread of a neurodegenerative tissue phenotype across anatomically connected brain regions [59]. Similarly, hepatitis B virus can employ the EV biogenesis pathway of its host cells to encapsulate its virions and facilitate their efficient transmission to susceptible new host cells via a transport mechanism mediated by the large hepatitis B surface antigen [60]. EV formation and release are essential for the release of these viral particles. This indicates that the mechanism represents a non‐canonical route of virus dissemination distinct from the classical viral budding mechanism of most viruses.

### 2.4 Utility of Host‐Derived EV Subpopulations for Disease Diagnosis and Evaluation

#### 2.4.1 Utility of hEV‐Biomarkers in Assessing Disease Severity and Treatment Response

Mounting evidence supports the use of hEVs as biomarkers for infectious disease diagnosis and prognosis applications since they carry molecular signatures that can detect infections with high sensitivity and assess their severity. For example, longitudinal monitoring of EV miR‐483‐3p and let‐7d‐3p levels in sepsis patients has demonstrated strong correlations with their clinical metrics, including their Sequential Organ Failure Assessment (SOFA) scores, markers of endothelial injury, and 28‐day mortality [87, 88]. Notably, changes in these EV miRNA levels often precede overt clinical deterioration, suggesting their potential utility for early risk stratification and timely therapeutic intervention. Specific plasma EV miRNA profiles have also been shown to distinguish sepsis patients who develop acute respiratory distress syndrome (ARDS) from those who do not progress to ARDS, and the EV miRNAs associated with sepsis‐associated ARDS in this study were implicated in the regulation of glycoprotein VI signaling pathways that influence platelet activation and vascular inflammation responses [89]. EV miRNA signatures may thus have significant utility for early diagnosis of sepsis patients who progress to worse disease outcomes to facilitate earlier interventions that can lead to improved patient outcomes.

Serum EVs that are enriched for specific surface markers have also been shown to correlate with the severity of COVID‐19 disease. One study reported that CD13^+^ and CD82^+^ EV subsets were markedly increased in patients with severe disease and associated with elevated myeloid‐derived suppressor cell levels and serum concentrations of pro‐inflammatory factors, consistent with immune dysregulation associated with severe disease [32]. Conversely, this study found that CD24^+^ EVs were inversely correlated with severe disease and myeloid‐derived suppressor cell levels, suggesting a protective or regulatory function. These dynamic changes in hEV subtypes with disease severity suggest their potential to serve as biomarkers of disease progression and the immune response during viral infections. EV surface proteins may thus also have strong potential to rapidly assess or predict disease severity to permit earlier intervention that may improve patient outcomes. Conversely, attenuation of EV biomarkers associated with disease severity may also provide an effective means to evaluate treatment response.

#### 2.4.2 Utility of hpEVs for Disease Diagnosis and Prognosis

EVs secreted by infected cells can carry molecular signatures derived from the host cell and its pathogen that can provide powerful advantages when employed for disease diagnosis and prognosis [90]. Several intracellular pathogens have evolved mechanisms that alter EV biogenesis in their hosts to ensure that their genomic material, mRNAs, proteins, or virulence factors are selectively packaged into the EVs secreted by these infected cells [16, 17, 19]. Several viruses can alter EV sorting signals or modify cell mechanisms to preferentially enrich secreted EVs with viral RNAs or proteins, whereas intracellular bacteria, fungi, or parasites may exploit host cell stress responses to enhance the packaging of pathogen‐derived nucleic acids and proteins into these EVs. This selective enrichment can enhance the detection of pathogen‐specific markers against the host background to permit more sensitive disease diagnosis during early infection or in complex clinical presentations that exhibit symptom overlaps. Particularly since EVs carrying these factors may be less subject to immune recognition and clearance than pathogen‐derived EVs, since they may express fewer surface antigens and display factors that attenuate their recognition and clearance by the innate immune system.

EVs secreted by infected cells can also carry host‐derived molecules (e.g., signaling factors, cytokines/chemokines, and other factors involved in the innate and adaptive immune response) that can provide useful information regarding the status of the pro‐inflammatory, immune, and tissue injury responses of the host organism that can be used to evaluate disease severity, progression, and the response to treatment. Integrated analysis of both host‐ and pathogen‐derived biomarkers can provide important information about interactions among an infection and its associated immune response, which is often critical for prognostic assessment and therapeutic decisions (Table 2). For example, specific patterns of pathogen‐ and host‐derived EV markers may distinguish acute and chronic infections or differentiate infections caused by pathogen subtypes with distinct virulence or resistance phenotypes to predict patient outcomes and inform treatment. Longitudinal profiling of hpEV biomarker signatures can also detect early signs of positive treatment responses or relapse to rapidly inform treatment decisions.

**TABLE 2:** Infectious diseases and hpEV‐associated biomarkers.

Disease	hpEV biomarker	Sample source	Detection platform	Clinical utility	Translational stage	Diagnostic performance	References
Tuberculosis	LAM‐associated EV proteins, mycobacterial RNA	Plasma	Immunocapture + PCR	Early diagnosis, treatment monitoring	Near‐clinical	Sensitivity ≈74%; detects microbiology‐negative cases; active versus latent discrimination (pilot)	[91, 92]
COVID‐19	Spike protein, viral RNA	Plasma	nanoFCM, dPCR	Disease stratification	Near‐clinical	Earlier and broader detection than plasma RT–qPCR; identifies RT–qPCR–negative cases	[93]
Sepsis	Bacterial antigens + host inflammatory miRNAs	Plasma, serum	Targeted EV miRNA qRT‐PCR array+ small RNA sequencing	Early risk stratification	Exploratory	Early discrimination and risk stratification (pilot)	[87, 89]
Hepatitis C	EV‐associated HCV RNA and host miRNAs (e.g., miR‐122)	uMSC culture supernatant–derived exosomes	NTA (NanoSight NS300) + WB (CD63/CD81)	Non‐invasive infection detection and monitoring	Exploratory	Indicates active replication and treatment non‐response (monitoring utility)	[75]
Malaria	Parasite proteins in plasma EVs	Plasma	Immunocapture‐based EV surface protein microarray	Non‐invasive diagnosis and stratification	Pilot clinical	High diagnostic accuracy for *P. falciparum* (AUC ≈0.97, pilot)	[92]
Fungal infection (*Candida*)	Fungal EV proteins and host response markers	Biofilm	LC–MS/MS–based EV proteomics with machine‐learning‐assisted analysis	Early infection detection and host–pathogen profiling	Exploratory	Exploratory virulence‐associated pEV biomarkers	[68]

Together, pEVs and hEVs provide complementary, yet incomplete, insights into infectious disease processes: pEVs primarily reflect pathogen‐centric signals, whereas hEVs encode host responses. This conceptual gap motivates the emergence of hpEVs as an integrative EV subtype with unique diagnostic potential, as discussed in the following section.

## 3 New and Current Methods Employed for hpEV‐Based Diagnostics

EVs secreted by pathogen‐infected host cells offer a promising source of host and pathogen‐derived biomarkers for minimally invasive infectious disease diagnostics. However, the heterogeneity and low abundance of diagnostic EV subsets present technical challenges, prompting the need for specialized tools and integrative analytical strategies. Against this backdrop, this section summarizes emerging analytical strategies for hpEV‐based diagnostics and their technological and translational implications.

### 3.1 Need for hpEV‐Based Diagnostics

A multifaceted diagnostic strategy can be necessary to analyze hpEV biomarker signatures due to the complexity of EV populations and compositions in most diagnostic specimen types. Biomarker discovery studies frequently employ proteomics and nucleic acid profiling methods to identify host‐ and pathogen‐derived factors that can distinguish hpEVs from other hEV populations and specific disease stages and phenotypes. However, these methods are not suitable for routine use in diagnostic applications. In the following subsections, we therefore describe several key assay approaches employed to detect disease‐associated EV NA and protein biomarkers, and how integrating these methods can improve diagnostic applications.

#### 3.1.1 Methods That Detect hpEV‐Associated Proteins

Proteomics provides a powerful means for the systematic analysis of proteins associated with hpEVs to facilitate the discovery of disease‐related biomarkers from a variety of specimen types, with EVs obtained using a variety of isolation procedures. For example, EVs can be isolated from the total EV population of non‐invasive patient urine samples by high‐throughput EV enrichment techniques such as ultrafiltration and analyzed by mass spectrometry to provide an unbiased profile of EV protein and lipid composition. This can permit simultaneous detection of pathogen‐ and host‐derived factors to support identification of potential diagnostic and monitoring biomarkers and provide valuable insight into processes associated with disease progression or host–pathogen interactions [94, 95]. Studies that employ specimens enriched in EVs secreted by a specific cell population can, however, provide more restricted datasets to facilitate the study of pathogen–host interactions. For example, mass spectrometry analysis of EVs isolated from hepatic blood samples of humanized mice infected with *Plasmodium vivax* detected host‐ and pathogen‐derived EV factors, which could potentially be used to identify specific infection stages [96], including asymptomatic malaria infections, to improve early disease diagnosis and surveillance efforts.

Proteomic analyses performed to identify EV biomarkers should evaluate whether identified proteins derive from the EV membrane or lumen since this determines which protein targets can be detected by specific methods. Bioinformatic approaches can often determine which proteins are associated with the EV membrane and luminal compartments, but it may also be necessary to segregate material from these compartments and perform separate proteomic analyses on them to detect low‐abundance proteins with high diagnostic significance and to confirm their localization. Such analyses require the use of approaches that can destabilize the EV membrane while minimizing the dissociation of transmembrane and membrane‐associated proteins (e.g., detergent treatment, freeze–thaw cycles, ultrasonic membrane disruption). Secondary analysis approaches may also be required to determine the facing of transmembrane and membrane‐associated proteins to confirm that identified protein biomarkers have outward‐facing regions that can be targeted by affinity probes.

Recognition of such surface biomarkers can be determined by a variety of techniques. This includes bead‐capture EV flow cytometry (FCM) and EV ELISA methods, although the latter approach can require an EV pre‐isolation procedure and lack sensitivity. Protein microarrays employing affinity factors have also enabled high‐throughput profiling of EV surface markers [97, 98, 99]. For example, a custom array of 24 capture antibodies printed on epoxysilane‐coated slides has been used to identify significant differences in the EV surface expression of CD81, CD106, HLA‐DR (human leukocyte antigen–DR), osteopontin, and other markers on EVs obtained from individuals with malaria versus healthy controls, indicating the potential utility of EV membrane protein signatures as diagnostic biomarkers [100]. A dark‐field microscope analysis approach that used a nanoparticle‐enhanced immunoassay to detect the *M. tuberculosis* virulence factors lipoarabinomannan (LAM) and its carrier protein LprG, on circulating EVs [91] (Figure 4A) was also able to distinguish active and latent tuberculosis cases in a non‐human primate model and detect active tuberculosis in pediatric cohorts with and without HIV co‐infection. A subsequent study employed a non‐disruptive method to generate monocrystalline copper shells on nanoparticle‐conjugated antibodies bound to these EVs to enhance the surface plasmon resonance from the resulting copper nanocubes [92] (Figure 4B). This approach yielded a 70‐fold increase in signal intensity and a 20‐fold improvement in the assay's limit of detection versus the original assay and outperformed standard assays used to diagnose tuberculosis in the study population.

**FIGURE 4:**
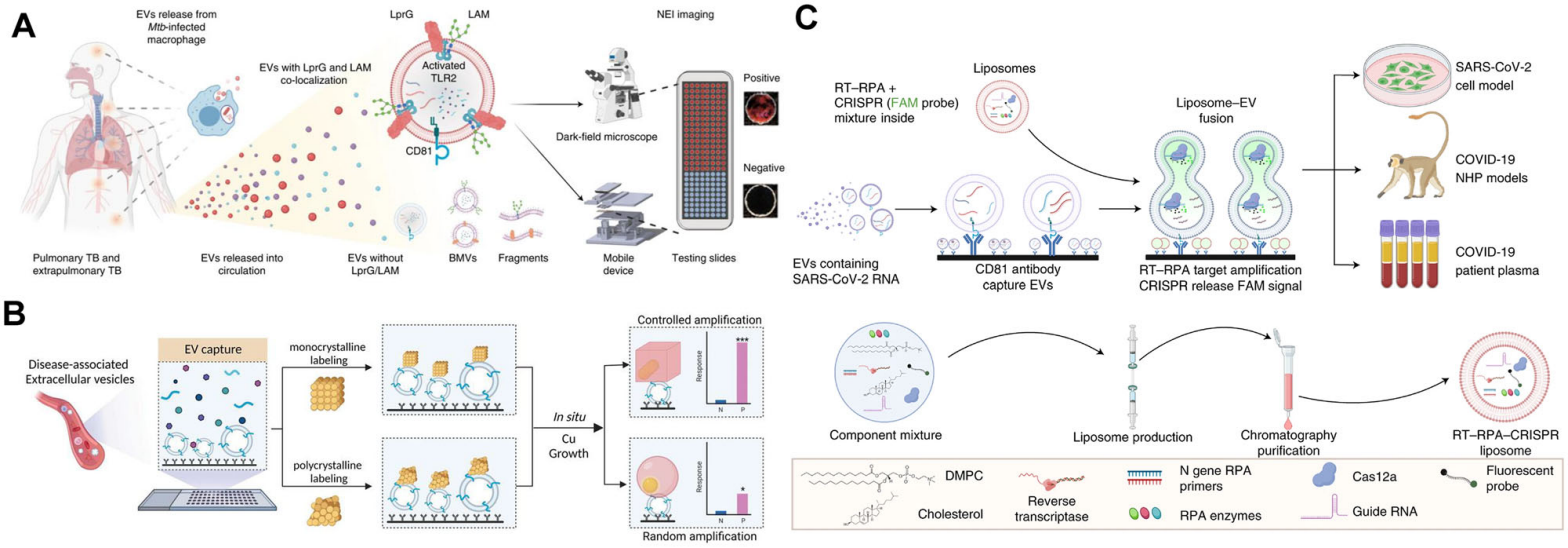
Detecting pathogen‐derived proteins and nucleic acids in hpEVs for infectious disease diagnostics. (A,B) Copper nanoparticle‐enhanced immunoassay (Cu‐NEI) detects Mycobacterium tuberculosis surface antigens (LAM and LprG) on hpEVs using dark‐field microscopy. (A) Reproduced with permission [91]. Copyright 2022, Springer Nature. (B) Reproduced with permission [92]. Copyright 2022, Wiley‐VCH. (C) Liposome–EV fusion enables fluorescent detection of intravesicular SARS‐CoV‐2 RNA via RT–RPA–CRISPR. Reproduced with permission [93]. Copyright 2021, Springer Nature.

Detection of target proteins within EV populations is more complex, since this requires the use of membrane permeabilization procedures or reporters or detection systems that can cross the EV membrane without disrupting or destabilizing it. In one example of the first approach, electroporation was used to transfer antibodies conjugated with quantum dots into EV samples to selectively visualize viral ribonucleoproteins within EVs [101], and identified a new mechanism where influenza A virus used EVs to evade immune detection. Reporter systems or probes can also be induced to cross the EV membrane using reagent‐loaded liposome fusion methods or reporters that can directly diffuse through this barrier [93, 102].

#### 3.1.2 Methods That Detect hpEV‐Associated Nucleic Acids

EVs also carry nucleic acids—predominantly RNA [103], with smaller quantities of DNA [104]—that can provide highly useful information about the specific host or pathogen phenotypes. However, like luminal proteins, simplicity of their analysis is limited by their presence behind the EV lipid bilayer [105]. EV nucleic acid analysis approaches thus usually require disruption of the EV lipid bilayer, necessitating that analyses be performed with either population‐level nucleic acid isolates or in volume‐limited digital droplet approaches. Some studies have used molecular beacon probes designed to penetrate the EV membrane for in situ nucleic acid detection as an alternative to droplet‐based nucleic acid detection, but this approach yields suboptimal efficiency [106, 107, 108]. However, we have reported that a liposome–EV fusion can permit sensitive in situ detection of viral RNA in plasma‐derived EVs (Figure 4C) [93]. In this streamlined assay approach, EVs are directly captured from plasma onto a detection surface using an antibody specific for an EV surface protein and then induced to fuse with liposomes carrying target‐specific reverse transcription‐recombinase polymerase amplification (RT‐RPA) reagents and a CRISPR–Cas12a reporter system that produces a fluorescent signal proportional to the target amplicon concentration. This method permits rapid, sensitive, and specific target detection while minimizing RNA degradation and off‐target effects, which could improve early‐stage disease diagnosis and rapid monitoring of disease responses to therapeutic interventions.

#### 3.1.3 Methods That Simultaneously Detect hpEV Protein and RNA Biomarkers

EV analysis approaches have traditionally focused on either protein or nucleic acid targets, and thus cannot assess the complex interplay between these biomolecules within specific EV populations or single EVs. This reductionist approach can limit our ability to understand protein and RNA biomarker associations that could indicate biomarker interactions associated with important cell signaling pathways and host–pathogen dynamics. Simultaneously profiling both proteins and RNA could thus enhance sensitivity, specificity, resolution, and diagnostic and prognostic accuracy of diagnostic applications for infectious disease [109, 110]. One promising high‐throughput approach employs single‐particle interferometric reflectance imaging (SP‐IRIS) with single‐molecule fluorescence in situ hybridization (smFISH) [111]. This SPIRFISH method permits simultaneous detection and quantification of specific proteins and RNA targets and has been used to detect HIV‐1 envelope protein (gp120) and genomic RNA (gRNA) in individual EVs, to distinguish infectious (gp120+/gRNA+) and non‐infectious (gp120+/gRNA‐) EVs derived from cells with active and latent HIV‐1 infections, respectively. Notably, detecting EVs expressing virus RNA/DNA or protein alone may not reliably detect active infections or virus transmissibility. EVs secreted by cells with both active and latent infections may express virus proteins, but EVs of the latter cells should not contain virus genomic material. Conversely, EVs secreted by cells infected with transcriptionally active and silent/defective viruses may both contain virus genomic material, but only the former may carry virus proteins [112, 113]. This dual analysis approach could thus provide a more reliable means to provide molecular information for disease diagnosis and clinical decision‐making for some viral diseases.

### 3.2 New Technologies With Potential for hpEV‐Based Disease Diagnostics

The sensitivity and specificity of current EV analysis approaches present a major limitation for hpEV diagnostics since hpEV biomarkers may be difficult to detect against a strong and complex background from other more abundant EV biomarker populations present in clinical specimens. This may be particularly problematic during early infection when hpEVs and hpEV biomarker levels may be present at very low concentrations and difficult to detect. New EV analysis methods employ signal amplification strategies, single vesicle‐resolution analyses, integrated microfluidic systems, and AI‐driven approaches to provide enhanced sensitivity, resolution, and multiplexing capabilities to address this issue and others [114]. Many of these techniques were originally developed for other biomedical applications but have shown strong potential for disease diagnostics—particularly for multiplex applications that simultaneously profile host and pathogen biomarker signatures within single EVs. Sensitive multi‐parametric EV analysis methods could substantially improve diagnostic sensitivity by capturing the full range of disease heterogeneity and by more accurately excluding related conditions to reduce false positives.

Integrating these advanced analytical platforms with standardized EV isolation protocols and clinically validated workflows will be key to unlocking the full potential of EV‐based applications for disease diagnosis and treatment evaluation. In the following sections, we provide a detailed overview of four research areas of particular interest—new signal amplification strategies, single vesicle‐resolution analysis methods, integrated microfluidic EV analysis systems, and AI‐driven data analysis methods—highlighting their underlying benefits, recent advances, and specific applications for EV‐based diagnostics (Table 3).

**TABLE 3:** Comparative overview of emerging EV analysis technologies.

Metric	Signal amplification strategies	Single‐vesicle resolution methods	Microfluidic platforms	AI‐driven approaches
Representative approaches	CRISPR‐based detection, TSA, TASs	SP‐IRIS, nanoFCM	Lab‐on‐a‐chip EV capture and analysis	Machine learning and multimodal data integration
Sensitivity	High	High	Moderate–high	Indirect (data‐driven)
Specificity	Moderate–high	High	High	Variable
Cost	Low–Moderate	Moderate–High	Low–Moderate	Low–Moderate
Scalability	High	Moderate	High	Very high
Key strengths	Strong sensitivity enhancement; broad compatibility with analytical platforms	Single‐vesicle characterization; high molecular specificity	Integration, automation, and portability; low sample consumption	Pattern recognition; scalable data interpretation
Limitations	Potential signal bias; limited quantitative accuracy	High cost; limited throughput	Device variability; limited standardization	Data dependency; limited interpretability
References	[115, 116, 117, 118, 119, 120, 121, 122, 123, 124, 125, 126, 127]	[128, 129, 130, 131, 132, 133]	[134, 135, 136, 137, 138, 139, 140]	[141, 142, 143, 144, 145, 146, 147]

#### 3.2.1 Signal Amplification Strategies

Infectious disease diagnostics now in use often lack the sensitivity necessary to detect latent infections or other infections that may produce low concentrations of pathogen‐associated biomarker signals. Adopting new signal amplification strategies to detect and analyze disease‐associated EVs could significantly improve the sensitivity of such assays, which should improve their diagnostic accuracy, permit earlier diagnosis, better detect latent infections, and enable use of less invasive diagnostic specimens that may contain lower biomarker levels than conventional specimens. Such assays could be particularly valuable in detecting hpEVs that carry low‐abundance pathogen‐derived nucleic acids if they permit them to be distinguished from the high nucleic acid background from the hEV population of a specimen.

A variety of techniques have been effectively employed for nucleic acid amplification, including PCR, hybridization chain reaction (HCR), and rolling circle amplification (RCA), with new add‐on approaches, such as CRISPR‐based diagnostics and Ago‐mediated systems, offering further sensitivity gains [115, 116, 117, 118, 119, 120]. Each method has distinct advantages when employed to amplify low‐abundance EV nucleic acid biomarker signals. HCR and RCA assays employ isothermal amplification to selectively increase the abundance of scarce biomarker targets in diverse backgrounds and are suitable for point‐of‐care applications. CRISPR‐based assay systems that employ Cas12a or Cas13a can be used to couple guide RNA‐mediated target recognition with an inherent collateral cleavage activity that rapidly cleaves an assay reporter to be sensitively read by either fluorescent or lateral flow assay readouts systems. Similarly, argonaute (Ago) assays primed with specific NA templates can also be employed to direct sequence‐specific recognition and cleavage of a target NA sequence. Both these systems can be combined with isothermal amplification approaches to enhance the sensitivity and specificity of NA target recognition, although each approach has specific advantages and disadvantages.

However, few strategies are available to enhance the sensitive detection of EV‐derived protein biomarkers, and most of these rely on the use of tagged affinity molecules that directly or indirectly amplify biomarker recognition events. These can primarily be divided into methods that employ affinity molecules conjugated with enzymes or NA target sequences. In the first case, enzyme‐ or nanozyme‐conjugated affinity molecules usually drive reporter conversion reactions that can be sensitively measured by optical readout systems. For example, tyramide signal amplification (TSA) and nanoparticle‐enhanced methods—including nanozyme systems with enhanced catalytic activity and nanoparticle‐enhanced Raman scattering methods—are commonly applied to increase protein detection signals [121, 122, 123, 124]. Recent approaches have also used NA‐labeled affinity molecules to convert target protein recognition events into NA signal outputs [148, 149, 150]. For example, one group employed a two‐aptamer system to detect EVs that expressed both epithelial cell adhesion molecule (EpCAM) and programmed death‐ligand 1 (PD‐L1) proteins on their surface membranes. In this method, EV membrane fluidity facilitated the proximity ligation of EPCAM and PD‐L1 aptamers bound on the same EV, and these ligation products were then detected by digital droplet PCR to directly quantify the number of EVs that expressed both proteins [149]. Similar assays could also be employed to detect hpEVs expressing specific combinations of host‐ and pathogen‐derived factors.

EV enrichment and isolation strategies that use antibodies specific to commonly expressed EV transmembrane factors are widely employed to separate EVs from other particles in diagnostic specimens but are expensive and difficult to scale. Some studies have replaced EV capture antibodies with aptamers to reduce EV isolation cost, while others have exploited the physical characteristics of EVs for their selective isolation. For example, EV membranes are selectively enriched in phosphatidylcholine (PC), phosphatidylserine (PS), phosphatidylethanolamine (PE), cholesterol, and sphingolipids [151], and lipoaffinity‐based EV enrichment strategies that exploit this difference have recently emerged as cost‐effective and scalable solutions for EV isolation [152]. Several lipoaffinity strategies have employed functionalized nanomaterials or engineered surfaces to capture high‐purity EV fractions while preserving EV membrane integrity [153], including studies that have used molecules that are structurally analogous to EV phospholipids. For example, one group used avidin‐coated magnetic particles to efficiently capture EVs after their incubation with biotin‐tagged 1,2‐distearoyl‐sn‐glycero‐3‐phosphoethanolamine–poly(ethylene glycol) (DSPE–PEG) [154]. A subsequent study employed a desthiobiotin‐labeled lipid to allow selective release of the lipoaffinity‐captured EVs upon incubation with free biotin, and then used a lectin‐induced aggregation approach to convert EV glycan expression into a size‐based signal high‐sensitivity readout for tumor diagnosis [155]. Other studies have employed a supramolecular probe composed of branched dendrimers functionalized with choline phosphate for EV enrichment since this probe forms electrostatic and hydrophobic interactions with membrane lipids and sterols, exhibits strong affinity for plasma membranes, and efficiently binds EVs [156, 157, 158].

EV enrichment and detection approaches can also be integrated into a single procedure to streamline biomarker detection and reduce the variance associated with multi‐step assay procedures. For example, one study directly labeled EVs captured from a biological specimen by using them as a substrate for the synthesis of DSPE–PEG‐modified gold nanozymes due to the strong affinity of DSPE for the EV lipid bilayer [159]. A second study employed a similar approach to synthesize choline phosphate (CP)‐grafted platinum nanozymes on captured EVs as a universal EV probe for a high‐throughput, high‐sensitivity immunoassay [160] (Figure 5A), since CP exhibits better antifouling properties than DSPE–PEG to yield greater EV specificity and lower background. Another integrated approach employs thermophoretic aptasensors (TASs), which have recently emerged as a promising technique for EV detection [125, 126, 127]. In this approach, a specimen is incubated with a fluorescently tagged aptamer probe that recognizes a target molecule on the EV surface and then illuminated with a 1480 nm laser to establish a highly localized temperature gradient, which causes all EVs to migrate toward the center of the laser spot (Figure 5B). This thermophoretic EV enrichment effect concentrates EVs into a restricted volume to enhance the signal density of the aptamer‐labeled EVs and thus assay sensitivity, and this thermophoretic approach can also be employed to detect EV RNA cargo.

**FIGURE 5:**
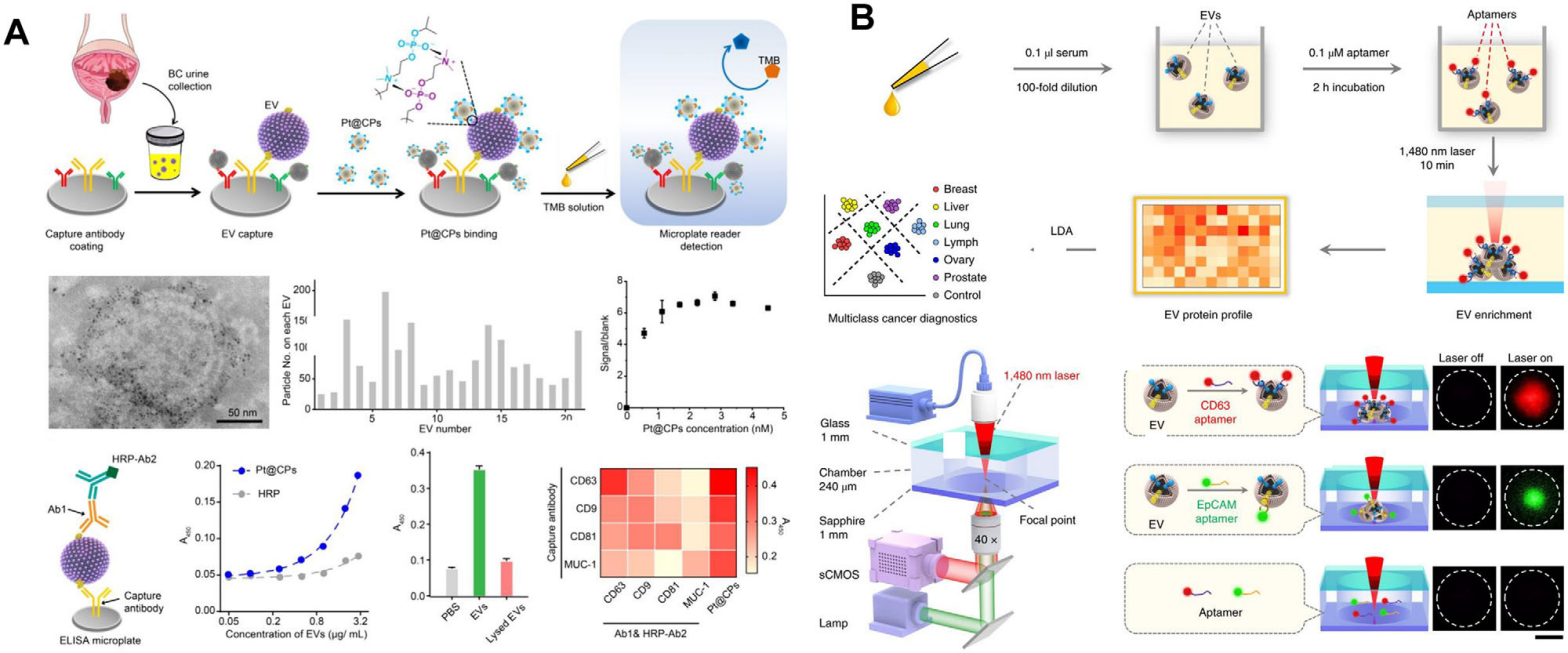
Signal amplification strategies for EV analysis. (A) Coordination polymer‐based substrates enable efficient capture of intact extracellular vesicles through multivalent surface interactions, thereby preserving native membrane structure and epitope accessibility. Subsequently, capture antibodies selectively immobilize bladder cancer‐associated EVs, after which platinum‐coordination polymer nanoprobes non‐specifically adsorb onto the EV lipid membrane to generate vesicle‐level signal amplification. As a result, each captured EV is converted into multiple catalytic reporting units, leading to enhanced sensitivity and lower limits of detection compared with conventional ELISA. Reproduced with permission [160]. Copyright 2024, American Chemical Society. (B) Infrared laser‐induced thermophoresis generates a localized temperature gradient that selectively enriches aptamer‐bound EVs within the detection zone, while free aptamers are excluded due to their small molecular size. This size‐dependent enrichment increases local EV concentration and enhances the signal‐to‐background ratio for sensitive EV detection. Reproduced with permission [125]. Copyright 2019, Springer Nature.

#### 3.2.2 Single Vesicle Resolution EV Analysis Methods

Given the heterogeneity of EV populations in diagnostic specimens and the need to distinguish sometimes rare pathogen‐associated subpopulations in these specimens, a consequence of diverse EV biogenesis pathways and non‐uniform cargo loading at the single‐vesicle level, it is often useful to employ methods that can analyze EVs at single vesicle resolution analysis to accurately detect specific EV biomarker signals with minimal background noise [34]. Conventional bulk EV analysis approaches detect specific EV biomarker target signals within heterogeneous EV populations, potentially masking signals from scarce yet biologically significant EV subpopulations [161]. Single‐EV analyses can thus profile molecular differences on individual EVs to provide more precise definition of biomarker signal distributions within an EV population that can provide new insight into disease processes [161, 162] to improve the sensitivity and specificity of future EV diagnostics [128] (Figure 6A). For example, one study has reported that single‐EV analysis of key cancer‐associated mutant proteins, including KRAS and P53 mutants, facilitated the detection of stage 1 pancreatic cancer [129] (Figure 6B) and detected pancreatic ductal adenocarcinomas of ≥0.1 cm^3^, surpassing the resolution of standard clinical imaging methods. Single EV analysis methods are, however, often constrained by technical challenges associated with detecting and resolving distinct biomarker signals derived from individual EVs due to the limited number of targets that can be displayed on or within these nanoscale particles. High‐resolution and super‐resolution microscopy techniques are most frequently used for single‐EV detection methods [23], since advances in instrumentation and probe chemistry now permit them to detect and resolve multiple fluorescent labels on distinct EVs [85, 128]. At least one study has also used a combined fluorescence and atomic force microscopy approach to evaluate the molecular composition and physical properties of target EVs [163]. Most of these high‐resolution microscopy approaches are both technically challenging and time‐consuming, rendering them better suited for biomarker discovery studies rather than diagnostic applications.

**FIGURE 6:**
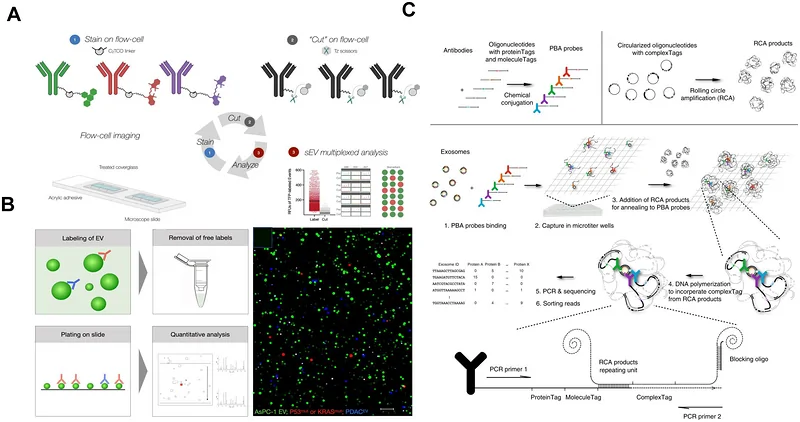
Single EV–resolution analysis. (A) Iterative multiplexed analysis of single EVs (MASEV) enables repeated staining, cleavage, and quantification to build biomarker profiles at single‐vesicle resolution. Reproduced with permission [128]. Copyright 2023, Springer Nature. (B) Single‐EV analysis (sEVA) uses protein‐reactive dyes and fluorescent antibodies for imaging‐based EV phenotyping. Reproduced with permission [129]. Copyright 2022, American Association for the Advancement of Science (AAAS). (C) Schematic of the PBA (Proximity Barcoding Assay) workflow. Antibody–DNA probes carrying unique protein and molecule tags are used to label surface proteins on individual EVs. Labeled EVs are captured via cholera toxin B (CTB), and probes bound to the same EV hybridize to a shared RCA product carrying a unique complex tag. Enzymatic extension incorporates this complex tag into the probe DNA, which is then selectively amplified and sequenced to profile protein identities at the single‐EV level. Reproduced with permission [164]. Copyright 2019, Springer Nature.

Flow cytometry (FCM) analysis methods can permit high‐throughput single‐particle analyses, unlike most other single EV analysis methods but require specialized nanoFCM instruments that can resolve particles down to 40 nm in diameter [130, 131, 132] since conventional FCM systems have ≈500 nm particle detection thresholds [131]. NanoFCM analyses are simpler, faster, and often more sensitive than microscopy‐based single‐EV analyses, which can improve effective phenotypic profiling of EV surface markers for disease diagnosis [131]. For example, one NanoFCM‐based EV diagnostic application that targets seven ovarian cancer biomarkers has demonstrated high diagnostic potential for rapid and noninvasive diagnosis and classification of ovarian cancer patients [133]. However, despite their advantages, nanoFCM analyses are still limited by the availability by the number of fluorophores that can be resolved by an nFCM system and the availability of biomarker specific antibodies with useful spectral properties.

Proximity‐dependent barcoding assays (PBAs) can address the reporter resolution issue of single‐EV analyses since they permit high‐throughput, multiparametric profiling of up to 38 surface proteins on individual EVs [164] (Figure 6C) by using antibody‐conjugated DNA barcodes generated through rolling circle amplification (RCA) to generate distinct micrometer‐scale DNA clusters. In this process, the NA tags of the bound antibody probes are hybridized to added RCA products, enzymatically extended with a sequence‐specific tag, amplified by PCR, and then sequenced to identify the number of amplicons with specific tag combinations, and thus the proteins co‐expressed on single EVs. Key advantages of PBA include its scalability, minimal sample input requirement, elimination of labor‐intensive EV purification steps, and applicability across a broad range of protein targets and vesicle sizes. PBAs thus represent a powerful tool for dissecting EV heterogeneity and advance our understanding of EV roles in health and disease [165, 166, 167]. Further, the microscale particles produced when EVs hybridize with these RCA PBA products can be resolved by standard FCM or fluorescent microscopy approaches, and their biomarker expression patterns can be determined by resolving the fluorescent spectra of their bound reporter oligonucleotides, unlike other single‐EV methods.

#### 3.2.3 Microfluidic Platforms

Microfluidic devices can also provide substantial advantages for EV analysis approaches by enabling the development of compact, automated, and sensitive diagnostic platforms. Such devices can integrate multiple assay steps on a single chip and perform precise fluid handling steps to reduce assay variations that can impact the sensitivity and reproducibility of assay results.

EV diagnostics that employ microfluidic workflows are also ideal for point‐of‐care tests that may be employed in a range of situations, including remote and resource‐limited settings where conventional laboratory infrastructure is unavailable but rapid and accessible molecular testing can improve disease diagnosis and management [168, 169, 170], which is particularly important given the small size, physical fragility, and overlapping biophysical properties of EV subpopulations that require gentle and precisely controlled separation conditions.

Microfluidic devices permit simple and efficient isolation of high‐purity EV isolates from processed blood samples [134, 135] (Figure 7A), but can also rapidly isolate EVs from whole blood, as reported for one integrated device that separates plasma from whole blood [136] (Figure 7B). Notably, this example enriches EVs from whole blood within 45 min using a streamlined centrifuge‐based workflow that requires no additional laboratory equipment and produces higher‐purity EV fractions with greater recovery rates than conventional methods. Similar microfluidic approaches could thus improve EV isolation procedures necessary for research and clinical applications.

**FIGURE 7:**
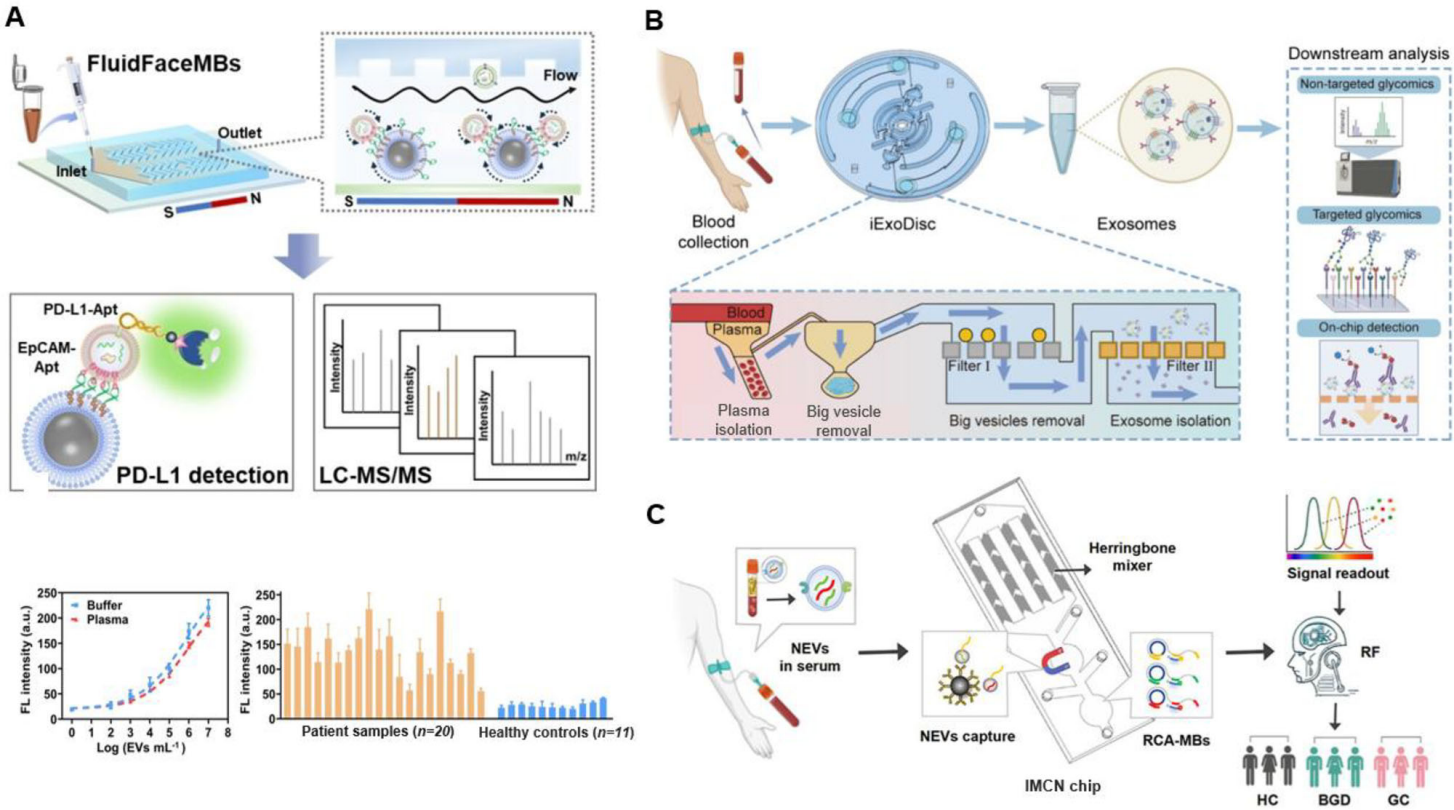
Microfluidic approaches for EV capture and disease biomarker detection. (A) Fluidic chip with a multivalent magnetic interface enables efficient capture, quantification, and PD‐L1 detection of tumor‐derived EVs from plasma. MB, magnetic beads. Reproduced with permission [134]. Copyright 2023, Wiley‐VCH GmbH. (B) iExoDisc enables on‐chip isolation, CD63‐based in situ detection, and point‐of‐care analysis of exosomes from whole blood for early cancer diagnosis. Reproduced with permission [136]. Copyright 2025, American Chemical Society. (C) Aptamer‐functionalized microfluidic chip allows magnetic field–assisted enrichment and multiplex analysis of EV‐associated neutrophil‐derived extracellular vesicles (NEVs) signatures, enabling accurate diagnosis and prognostic evaluation of gastric cancer when combined with conventional biomarkers. Reproduced with permission [137]. Copyright 2025, American Chemical Society.

Microfluidic platforms can also improve EV analysis by permitting multiple biomarker analyses to be performed in parallel on EV fractions with distinct physical or molecular characteristics [137] (Figure 7C). In one example, a device that uses a surface‐enhanced Raman scattering (SERS) signal readout has been reported to simultaneously detect multiple EV biomarkers in parallel [138] to detect biomarker expression levels and vesicle phenotypes that distinguish EVs derived from diseased and non‐diseased cells. Microfluidic devices can also facilitate the development of point‐of‐care assays that produce optical signals that can be detected and analyzed by compact readout devices, including smartphones [171]. For example, one group developed a smartphone‐readable assay that integrated a temperature‐ and redox‐responsive hydrogel matrix and a metamaterial with tunable mechanical properties to produce a shape‐transforming chiral interferometer that could sensitively detect target biomolecules [172]. In this approach, the assay system was first locked in a hyper‐responsive state by an LED‐activated plasmonic heating step so that target biomolecules that bound to hydrogel‐embedded antibodies produced rapid localized crosslinking events that altered the metamaterial's structure, changing its mechanical deformation to produce a diffraction pattern change detectable by a smartphone camera.

Microfluidic approaches have also been used to integrate EV separation, purification, and detection methods onto single devices [136, 139]. In one such case, a group developed a size‐coded affinity microbead system augmented by an in situ rolling circle amplification (RCA) strategy to achieve a fully integrated microfluidic design. This approach involved generating a library of color‐ and size‐coded microspheres by introducing quantum dots of varying colors into microspheres of distinct sizes [140], and then microgrids of matching dimensions were used to pre‐sort these microspheres on the microfluidic device to generate position‐specific signals without spectral overlap. EVs were then incubated with aptamers off‐chip, loaded onto the microfluidic device, and captured by hybridization of the extended aptamer sequence region with MJ5C‐L and SYL3C‐L hairpin probes linked to these microspheres to facilitate in situ RCA for signal amplification and measurement of specific biomarker‐positive EV populations.

#### 3.2.4 AI‐Driven EV Analysis Approaches

Artificial intelligence (AI) methods, including machine and deep learning‐based classification algorithms, have demonstrated substantial value in biomedical research studies by permitting the efficient analysis of high‐dimensional, multiparameter datasets and exhibiting robust ability to handle data with substantial noise or missing values [173, 174, 175]. These AI methods can facilitate the analysis of proteomic and genomic/transcriptomic data by capturing complex, nonlinear relationships to discover latent patterns in biological systems [141, 142].

EV datasets, which often comprise one or more of these data types and are characterized by high dimensionality and molecular complexity due to heterogeneous EV biogenesis, cargo loading, and the presence of both host‐ and pathogen‐derived signals within hpEVs, are therefore well‐suited for machine learning–driven biomarker discovery, EV subtype classification, and predictive modeling of disease mechanisms.

Several recent studies have demonstrated the promise of AI‐driven approaches for precision diagnostics. An EV‐based multi‐omics profiling approach has been reported to enhance the sensitivity and specificity of early cancer detection and provide mechanistic insights into EV roles in the pathogenesis of metabolic dysfunction‐associated steatotic liver disease [143] (Figure 8A). AI can also aid in the interpretation of optical spectroscopy datasets that capture the spatial and spectral characteristics of EVs [176, 177, 178, 179]. AI approaches are also particularly well‐suited to analyze spectral datasets, such as Raman spectroscopy data, that cannot feasibly be analyzed by other methods since AI algorithms excel at deciphering the complex, high‐dimensional data present in these datasets to facilitate rapid and accurate interpretation of EV signatures [180].

**FIGURE 8:**
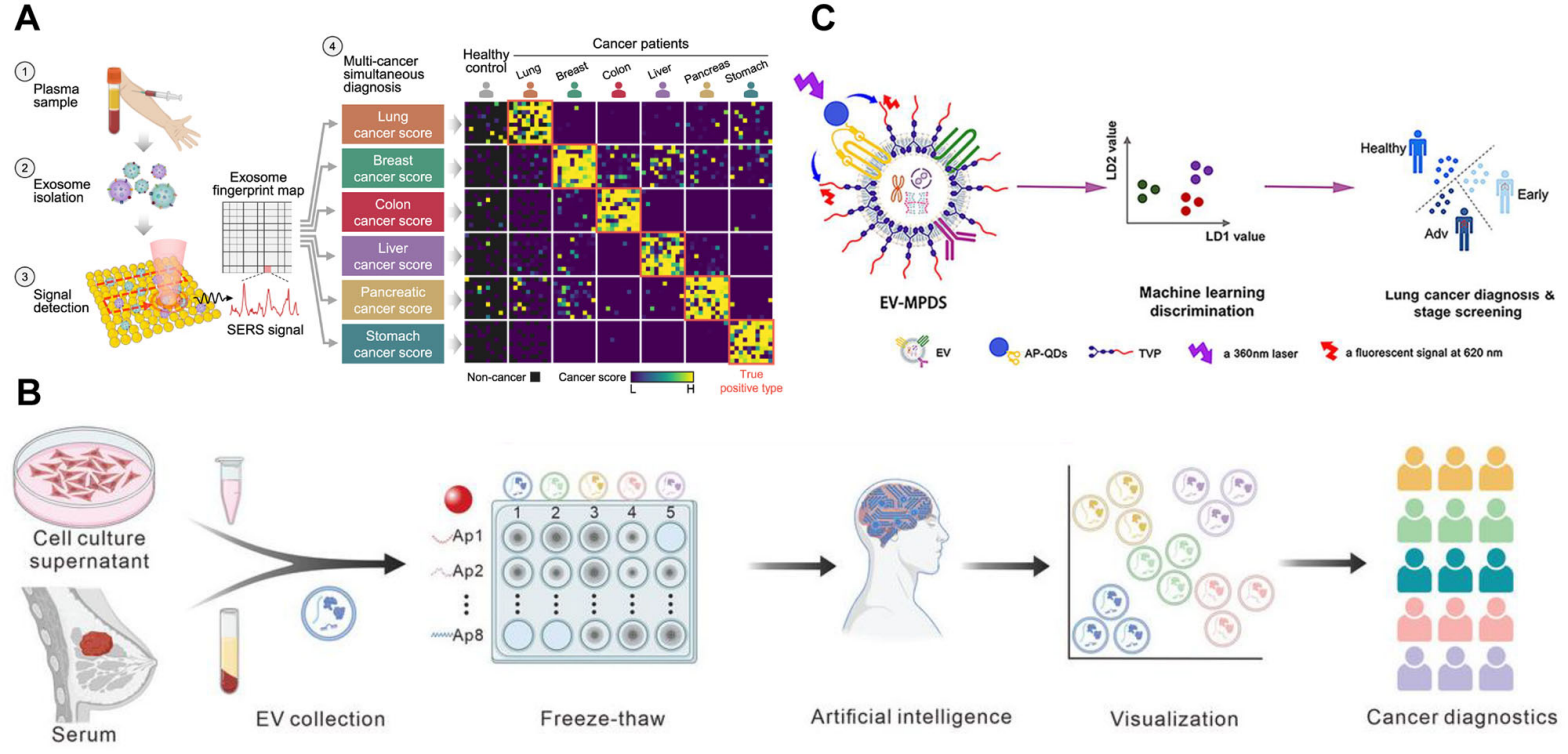
AI‐driven platforms for EV‐based cancer detection and tumor origin classification. (A) AI analyzes spatially mapped SERS signals to classify cancer type. Reproduced with permission [143]. Copyright 2019, Springer Nature. (B) Freeze–thaw‐induced floating patterns of gold nanoparticles are applied to clinical EV samples and analyzed using AI algorithms for cancer diagnosis. Reproduced with permission [177]. Copyright 2024, Wiley‐VCH GmbH. (C) EV‐MPDS (extracellular vesicle membrane protein detection system) utilizes Förster resonance energy transfer (FRET) for multiplex EV biomarker detection and is integrated with AI analysis to enable accurate lung cancer diagnosis and staging. Reproduced with permission [147]. Copyright 2023, American Chemical Society.

In one study, an AI‐enhanced SERS approach enabled label‐free analysis of EV‐derived spectral signatures, allowing deep learning‐based discrimination between cancer and healthy specimens, and inference of EV tissue of origin [144, 145]. Other studies have applied AI‐based pattern recognition to analyze optical signatures generated by EVs deposited on gold nanoparticle monolayers, successfully classifying breast cancer [146, 177] (Figure 8B). Building on this concept, another group developed an EV membrane protein detection system (EV‐MPDS) using quantum dot‐labeled aptamers together with a lipid dye to generate fluorescence resonance energy transfer (FRET) profiles of multiple lung cancer‐associated EV surface biomarkers. Coupling machine learning with these multi‐target optical spectra improved diagnostic accuracy, including for early cancer screening [147] (Figure 8C).

Collectively, these studies highlight AI's potential to extract clinically meaningful information from EVs' optical spectra for next‐generation diagnostics.

## 4 Challenges for Clinical Translation of hEV‐, pEV‐, and hpEV‐Based Diagnostics

Despite these technological advances, several factors hinder the clinical translation of EV‐based diagnostic applications. These include EV isolation challenges, a lack of standards and regulatory guidance, and cost and scalability issues. New efforts to overcome these challenges will be essential to advance EV‐based diagnostic tools from research settings to clinical practice [181, 182]. In this section, we discuss the core challenges facing EV‐based diagnostics and potential solutions to move the field toward clinical use (Figure 9).

**FIGURE 9:**
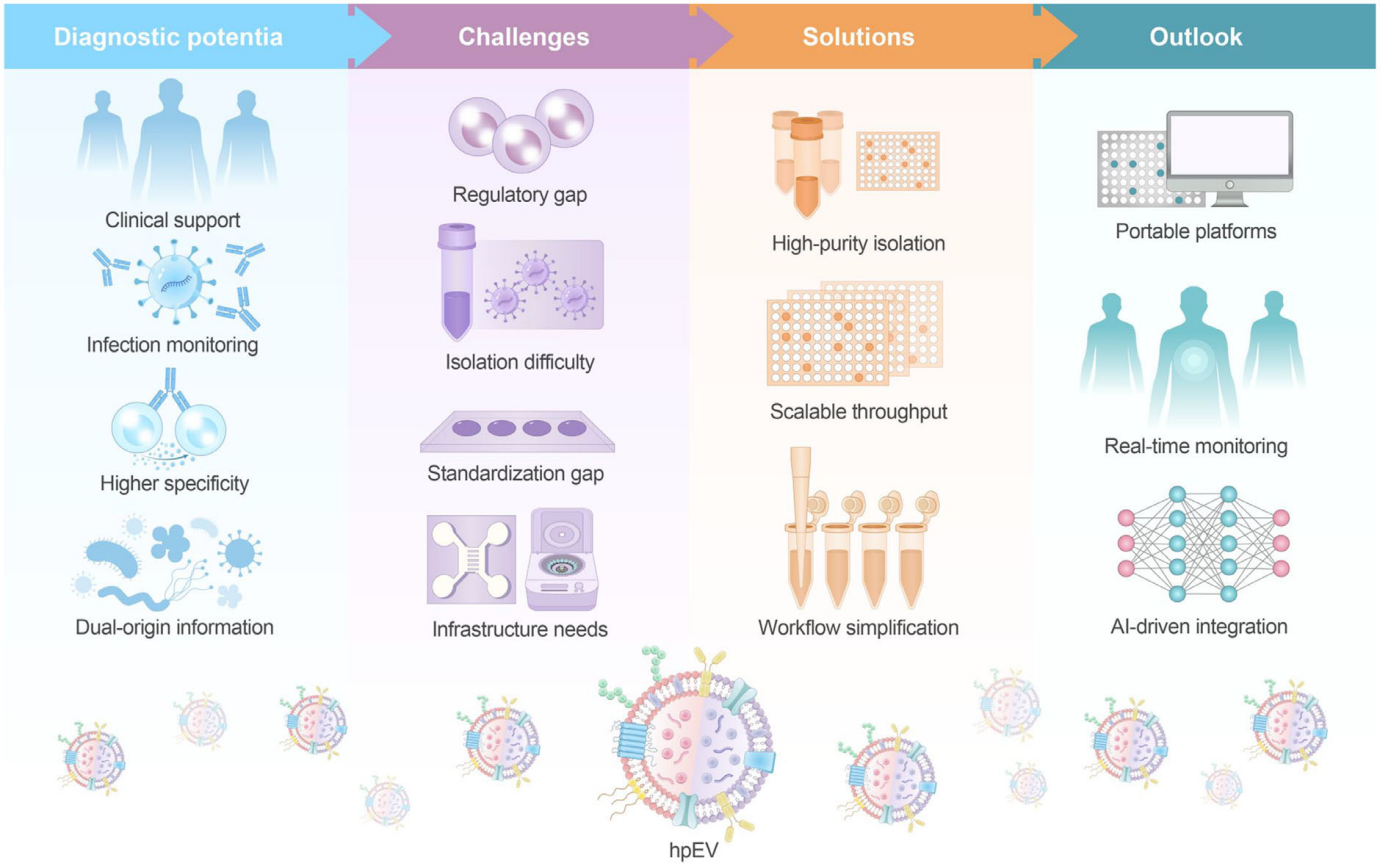
Translational challenges and potential solutions for hpEV‐based diagnostics. Barriers such as complex isolation procedures, regulatory uncertainties, and standardization gaps currently limit clinical implementation. However, advances in microfluidics, scalable high‐throughput systems, and decentralized point‐of‐care technologies provide promising strategies to overcome these hurdles and facilitate the integration of hpEVs into infectious disease diagnostics.

Importantly, the barriers to clinical translation of EV‐based diagnostics operate on different timescales. Near‐term challenges, such as pre‐analytical variability, EV isolation efficiency, and assay turnaround time, can be addressed through methodological optimization and platform integration. In contrast, longer‐term barriers—such as standardization, regulatory alignment, and large‐scale clinical validation—require ongoing, coordinated efforts across academia, industry, and regulatory bodies. Distinguishing these short‐ and long‐term challenges is essential for setting realistic development priorities and translational pathways.

### 4.1 EV Isolation Challenges

Reproducible isolation of high‐purity EV samples from diagnostic specimens or other biological samples remains a major technical barrier for the adoption of EV‐based assays [183, 184]. Representing a primary near‐term bottleneck for clinical translation. Standard EV isolation methods such as ultracentrifugation or size‐exclusion chromatography are widely used but suffer from limitations including their time‐consuming protocols, the co‐segregation of other material with their EV isolates, and their inability to resolve EV subtypes with overlapping sizes and biophysical properties. The purity of EV fractions produced by this method can be a particular issue since EVs usually co‐segregate with non‐vesicular factors such as proteins, nucleic acids, and lipoproteins when they are isolated based on their physical properties, which can mask biomarker detection or concentrate factors that may promote the degradation of accessible EV surface biomarkers or destabilize EV membrane integrity. EV integrity is also a significant concern for centrifugation and size‐fractionation methods as these may exert loading or shear forces that can disrupt their membrane integrity to reduce their stability or result in biomarker degradation or losses. In addition to isolation‐induced stress, EV integrity and recovery are also highly sensitive to pre‐analytical handling and storage conditions. Prolonged storage at −80°C and repeated freeze–thaw cycles can irreversibly degrade EV quality, reducing recovery, increasing particle size, elevating levels of co‐isolated contaminants, and causing membrane disruption or vesicle fusion [185]. These effects worsen with longer storage duration and more freeze–thaw cycles, underscoring the importance of using fresh specimens whenever possible or rigorously standardizing storage conditions and minimizing freeze–thaw events in EV‐based diagnostics. Both issues can reduce sensitivity and introduce variability to compromise diagnostic performance.

Several new EV isolation methods address these and other issues while providing additional benefits, such as reducing the amount of sample required for EV isolation, reducing workflow steps and complexity, and permitting the integration of EV isolation with analysis workflows [186, 187]. However, most of these new approaches are optimized to isolate EVs with defined physical or biochemical properties—not both—and are capable of limited throughput and lack robust standardization and clinical validation studies. This can be of significant concern when using these approaches to isolate rare hpEVs that may exhibit only minor differences from more abundant hEVs in the same specimen. Highly specific molecular probes may thus be required for sensitive and specific analysis of biomarkers associated with a low abundance hpEV population. Reliably isolating or resolving hpEVs from hEVs could provide additional insight into disease stage, the immune response, and therapeutic efficacy [188]. EV analyses performed at the EV subtype‐level may also help clarify how hpEVs and EVs influence disease‐associated processes to facilitate the discovery of new biomarkers and candidates for therapeutic intervention.

### 4.2 Lack of EV Standards and Regulatory Guidance

Ultimately, future progress in the clinical translation of EV‐based diagnostic applications will require the development of standard, clinically validated EV isolation methods capable of high throughput and reproducibility, highlighting the lack of standards and regulatory guidance as a long‐term structural barrier to widespread clinical adoption. Standard operating protocols for diagnostic specimen collection, handling, storage, and pre‐analytical processing procedures have yet to be established for EV research. This continues to impede the reproducibility of results generated by different groups, and the feasibility of performing multi‐center validation studies, which are required for the development and clinical translation of new EV applications. This has resulted in a growing gap between research findings and new clinical applications [189].

Cross‐study comparisons and regulatory approval are also hindered by the absence of standardized protocols for EV isolation, characterization, and biomarker validation. There is currently no established framework from major regulatory bodies such as the U.S. Food and Drug Administration (FDA) or the European Medicines Agency (EMA) to guide the clinical implementation of EV‐based diagnostics [190]. This regulatory uncertainty also hinders the development of standardized workflows necessary for clinical adoption. To address these challenges, academic institutions, industry stakeholders, and regulatory agencies must collaborate to establish consensus‐driven guidelines. The development of validated protocols, reference materials, and shared databases could facilitate regulatory alignment and accelerate clinical translation.

### 4.3 Cost and Scalability Issues

Practical constraints such as cost and scalability further impede the widespread integration of EV‐based diagnostics [191], posing a medium‐ to long‐term translational constraint, particularly for decentralized and resource‐limited settings. Many existing isolation and detection techniques rely on sophisticated instrumentation, limiting their accessibility in resource‐limited settings where some infectious diseases are most prevalent. The high cost of equipment and infrastructure demands of EV isolation and analysis workflows also limits their feasible use in decentralized or resource‐limited settings where EV diagnostics could have the greatest public health impact. New scalable, high‐throughput EV isolation and analysis strategies will be required to address these limits to the clinical translation of future EV assays. Many common EV isolation and analysis techniques (e.g., ultracentrifugation, nFCM, and mass spectrometry) require expensive equipment operated by highly trained personnel, and alternate methods are thus being developed to minimize these demands. These include nanoparticle‐enhanced lateral flow devices and smartphone‐based imaging systems that have the potential to provide low‐cost, high‐throughput, and point‐of‐care EV assay results. However, continued innovation will be essential to ensure that EV‐based diagnostics are not only biologically informative but also scalable, accessible, and practical for widespread use.

### 4.4 Emerging Clinical and Translational Progress of EV‐Based Diagnostics

Emerging clinical and translational progress in EV diagnostics is bridging the gap between experimental discovery and clinical implementation. Several clinical trials utilizing EV‐based biomarkers for disease detection, such as early cancer diagnostics and systemic disease monitoring, are currently registered in global clinical trial databases, highlighting active efforts toward clinical validation. Concurrently, commercial translation is underway, exemplified by companies such as exosome diagnostics, which have advanced EV‐based liquid biopsy assays (e.g., the ExoDx Prostate Test) for non‐invasive cancer risk assessment.

A key yet underexplored aspect of EV‐based diagnostics is detection timing relative to infection onset. Conventional antibody‐based tests typically exhibit a diagnostic window due to their reliance on adaptive immune response, which can delay detection in early or asymptomatic infection. In contrast, hpEVs may capture both early pathogen activity and immediate host cellular responses, offering the potential for earlier detection. However, direct head‐to‐head comparisons of hpEV‐based assays and antibody‐based tests with respect to time‐to‐detection remain limited, representing an important future research gap for longitudinal clinical studies.

These early clinical and commercial advances in EV‐based diagnostics are supported by a broader translational ecosystem driven by progress in EV‐based therapeutics. While most registered clinical trials currently focus on EV‐based therapeutics rather than diagnostics, these efforts establish a critical translational foundation for EV‐based biomarker development, including scalable manufacturing pipelines, standardized EV characterization frameworks, and emerging regulatory pathways that are equally essential for clinical validation and deployment of diagnostic assays [192].

Importantly, progress is further supported by coordinated standardization initiatives led by the International Society for EVs (ISEV), including the MISEV guidelines and the EV‐TRACK knowledge base, which promote harmonized workflows and transparent reporting [193]. Together, these initiatives reduce key translational barriers related to reproducibility, cross‐study comparability, and regulatory compliance, accelerating the clinical implementation of EV‐based diagnostics.

## 5 Conclusion

EVs play important roles in the development and progression of infectious disease through their role in mediating pathogen and host cell‐to‐cell communication events, and they also contain biomarkers that reflect this interplay between pathogen and host processes that regulate infection severity. This EV information can detect infections that are missed by conventional methods, including latent or asymptomatic parasitic, bacterial, viral, or fungal infections. EVs are therefore emerging as an important source of diagnostic and prognostic biomarkers for infectious disease, as they can carry host‐ and pathogen‐derived signatures that can sensitively detect an infection and the corresponding host response. Specifically, hEVs provide information about immune status and tissue injury; pEVs carry microbial factors with diagnostic specificity but have limited longevity; and hpEVs uniquely integrate these complementary features. Because hpEVs carry molecular signals derived from both pathogen activity and host responses, they provide integrated biological information not available through conventional pathogen‐ or host‐based assays alone. Rather than replacing existing diagnostics, hpEV‐based approaches complement them by offering a dynamic readout of ongoing host–pathogen interactions, with potential utility for distinguishing infection states, tracking disease progression, and monitoring treatment response, especially in settings where conventional diagnostics lack sensitivity or miss subclinical or heterogeneous disease processes [25].

EV‐based diagnostics are now poised to deliver rapid and scalable clinical assays that use diagnostic samples collected by minimally invasive methods due to the recent development of advanced signal‐enrichment and amplification procedures [194], high‐resolution single‐EV analysis methods, integrated microfluidic platforms for EV isolation and analysis, and AI‐driven pattern‐recognition algorithms. Notably, recent advances in microfluidic “lab‐on‐a‐chip” architectures now enable rapid, immunocapture of specific EV subtypes from just a few microliters of plasma, which can be seamlessly analyzed using downstream nanoFCM or label‐free optical readout methods. Microfluidic modules that employ dielectrophoresis or centrifugal “lab‐on‐a‐disc” systems can further automate sample processing and integrate it with analytic approaches to deliver true sample‐in/answer‐out workflows. Rugged and portable immunosensors or fluorescence and Raman spectra readout devices could also be employed to develop field‐deployable assays. Incorporating machine‐learning‐derived classifiers and real‐time cloud connectivity into these devices could also allow high‐density and multi‐omic EV data to be distilled into actionable clinical results—by detecting asymptomatic hypnozoite carriers, predicting therapeutic response in children with tuberculosis, or stratifying COVID‐19 patients by their risk for long‐term complications [195]. The development of fully integrated, portable diagnostic platforms capable of EV isolation, multiplexed signal acquisition, and embedded data analysis represents a critical frontier in infectious disease surveillance. New microfluidic [196], nanotechnology, and digital analytic approaches promise to democratize EV‐based diagnostic applications by enabling their decentralized use for real‐time monitoring of infectious disease in resource‐rich and resource‐limited settings. However, standardized pre‐analytical protocols, large‐scale clinical validation, and clear regulatory pathways will be essential to permit clinical translation of research findings to clinical applications that are required to achieve this goal.

Notably, the integration of new EV assays with AI‐driven classifiers and cloud‐enabled data integration methods promises to deliver actionable clinical information that could be used to detect latent or asymptomatic infections or stratify infected individuals according to their therapeutic responses or long‐term risks. Fully integrated diagnostic platforms that combine EV isolation, multiplexed signal acquisition, and embedded analytics thus address new frontiers in infectious disease surveillance, precision diagnostics, and personalized medicine. However, standardization of pre‐analytical workflows, large‐scale clinical validation studies, and clear regulatory pathways will be required to translate these advances into robust and widely accessible clinical applications.

## Author Contributions

Ming Zhou, Yongchun Pan, Bo Ning, Christopher J. Lyon,Yang Liu and Tony Hu contributed to the conceptualization. Ming Zhou and Yongchun Pan drafted the manuscript. Ming Zhou, Yongchun Pan, Bo Ning, Christopher J. Lyon, Yang Liu and Tony Hu provided critical revisions. Tony Hu and Christopher J. Lyon provided the funding support. Tony Hu was responsible for the decision to submit the manuscript.

## Conflicts of Interest

The authors declare no conflicts of interest.
